# RNA-inspired phosphate diester dynamic covalent networks†

**DOI:** 10.1039/d3py00867c

**Published:** 2023-09-01

**Authors:** Roy Wink, Soumabrata Majumdar, Rolf A. T. M. van Benthem, Johan P. A. Heuts, Rint P. Sijbesma

**Affiliations:** a Department of Chemical Engineering & Chemistry, and Institute for Complex Molecular Systems, Eindhoven University of Technology P.O. Box 513 5600 MB Eindhoven The Netherlands r.p.sijbesma@tue.nl j.p.a.heuts@tue.nl; b Department of Chemical Engineering & Chemistry, Laboratory of Physical Chemistry. Eindhoven University of Technology P.O. Box 513 5600 MB Eindhoven The Netherlands; c Shell Energy Transition Center Amsterdam Grasweg 31 1031 HW Amsterdam The Netherlands

## Abstract

Neighboring group assisted rearrangement substantially increases relaxation rates in dynamic covalent networks, allowing easier (re)processing of these materials. In this work, we introduce a dynamic covalent network with anionic phosphate diesters as the sole dynamic group, incorporating β-hydroxy groups as a neighboring group, mimicking the self-cleaving backbone structure of RNA. The diester-based networks have slightly slower dynamics, but significantly better hydrolytic (and thermal) stability than analogous phosphate triester-based networks. Catalysis by the β-hydroxy group is vital for fast network rearrangement to occur, while the nature of the counterion has a negligible effect on the relaxation rate. Variable temperature ^31^P solid-state NMR demonstrated a dissociative bond rearrangement mechanism to be operative.

## Abstract 2

Phosphate diesters were used as reactive moieties in dynamic covalent networks, harnessing internal catalysis by a neighboring β-hydroxy group. These networks are more hydrolytically stable than their phosphate triester counterpart.

## Graphical abstract

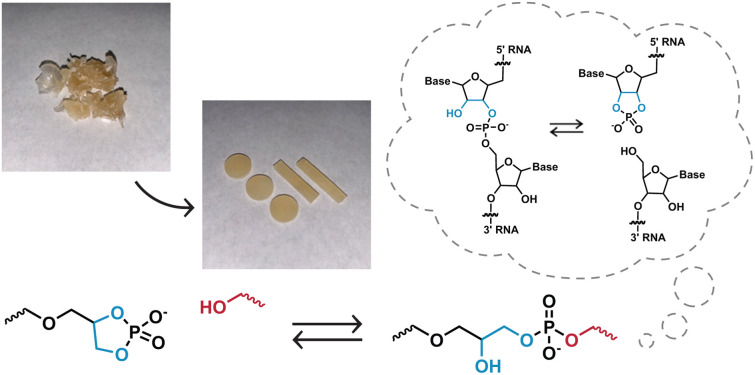

## Introduction

Dynamic covalent networks (DCNs) – also termed covalent adaptable networks (CANs) – are a rapidly developing class of covalently crosslinked polymers that can undergo network rearrangements through exchange reactions under external stimuli such as light or heat, thus allowing for flow in the material.^1–5^ Thermally activated DCNs have mechanical properties which are comparable to those of a thermoset at low temperatures, while at elevated temperatures, they are reprocessable like thermoplastics. Properties of these materials are to some extent based on the mechanism of rearrangement in the network, which is either associative or dissociative. For dissociative DCNs, bond dissociation precedes bond formation, resulting in a less crosslinked intermediate state. Whereas for the associative DCNs, new bond formation precedes bond dissociation, practically retaining crosslink density during network rearrangement.^5,6^ The concept of re-mendable polymers was first based on (retro) Diels–Alder reactions, where cross-links were reversibly broken at elevated temperatures *via* a dissociative mechanism.^7,8^ The first associative DCN, also called vitrimer, was introduced in 2011 by Leibler and co-workers, employing transesterification in a polyester-polyol network.^9^ Since then, a variety of chemistries has been introduced into the field of DCNs.^10–13^ The exploration of polymer networks combining dynamic covalent and non-covalent bonds has also been pursued.^14,15^ Recently, our group introduced phosphate triesters as dynamic moiety for catalyst-free associative DCNs.^16^ Network rearrangement in this system was based on transesterification, and the material showed stress relaxation properties at high temperatures comparable to carboxylate networks, yet without the use of a catalyst. Incorporation of phosphate esters could possibly provide properties like biodegradability,^17^ antifouling characteristics,^18^ and flame retardancy.^19^ Furthermore, phosphate esters are widely found in the chemistry of life,^20,21^ and are thus of interest for possible biomedical applications.

There is a growing interest in integrating DCNs in fast and industrially significant processing methods, such as (reactive) extrusion,^22^ melt blowing,^23^ and additive manufacturing methods like 3D-printing and direct ink writing.^24–26^ Dissociative DCNs are particularly suitable for these methods, by virtue of the decreased crosslink density and viscosity of these materials at processing temperatures.^27^ Still, many chemistries demand the implementation of an external catalyst in order to display exchange rates rapid enough for fast reprocessing. These catalysts may leach out over time, compromising long-term dynamic properties.^11^ Recent research by our group and others has therefore focused on DCNs with immobilized internal catalysts by means of neighboring group participation.^28–32^ In these DCNs, neighboring group participation leads to a dissociative rearrangement mechanism *via* cyclic intermediates that have a high energy, and are therefore present at low concentration, thus, just like vitrimers, the material does not lose its network integrity upon heating.

**Scheme 1:**
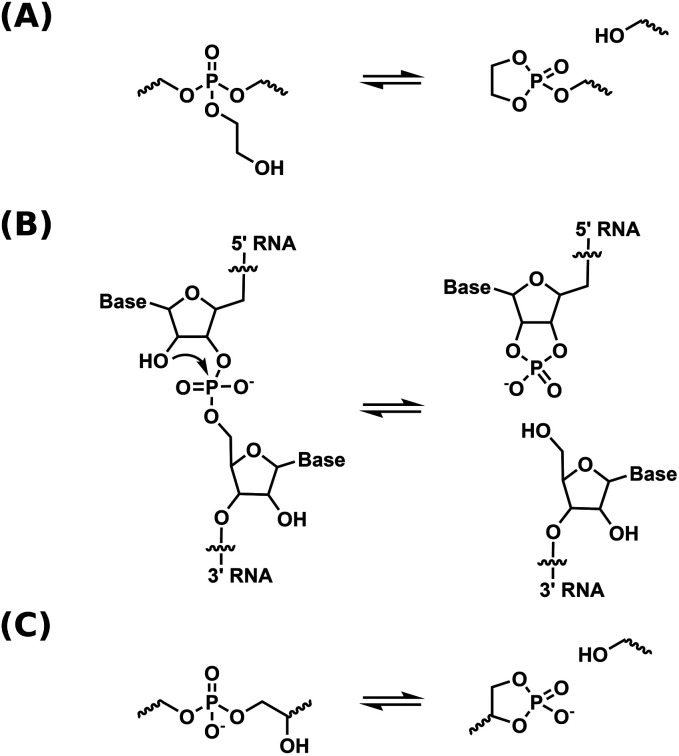
Schematic representation of a dissociative transesterification reaction in (a) phosphate triester DCNs.^33^ (b) RNA self-cleavage.^36^ (c) phosphate diester DCNs.

Our group has utilized neighboring group assisted rearrangement in phosphate triester-based dynamic covalent networks by means of pendent β-hydroxy groups.^33^ This β-hydroxy group allowed for the initiation of neighboring group mediated transesterification exchange reactions in the network *via* the formation of a cyclic phosphate triester intermediate (Scheme 1a). Stress relaxation rates were shown to be high enough for processing by extrusion. Variable temperature solid-state NMR spectroscopy was utilized as a method to prove a dissociative exchange mechanism. Although relaxation rates are fast, both the thermal- and hydrolytic stability limit the range of potential applications.

In contrast to this, phosphate diesters, especially in their anionic form, are hydrolytically more stable, but not dynamic enough for use in DCNs. The higher negative charge density on the phosphorus center makes for a less electrophilic moiety, and transesterification as well as hydrolysis are slow.^34^ The accelerating effect of neighboring group participation of the β-hydroxy group in phosphate diesters is a well-known concept in nucleic acid chemistry, where a β-hydroxy group (the 2′-OH of the neighboring ribose unit, see Scheme 1b) of RNA initiates self-cleavage, yielding a cyclic phosphate diester as cleavage product.^35,36^ Using β-hydroxy containing phosphate diesters in DCNs is therefore a promising approach to obtain dynamic, yet hydrolytically stable materials. The usage of β- and γ-hydroxy containing phosphate esters has been reported by other groups,^37,38^ but mechanistic aspects of diester transesterification in these materials were not studied in detail. Furthermore, the used synthetic method is known to yield a mixture of phosphate mono-, di- and triesters, complicating mechanistic studies.^39,40^

In the current work, we designed a DCN based on β-hydroxy containing anionic phosphate diesters as the sole dynamic moiety, closely mimicking the RNA backbone structure and its neighboring group assisted cleaving mechanism (Scheme 1c). To obtain these networks, a linker was made in two facile synthetic steps, and combined with a polar or an apolar polymer backbone to yield two dynamic polymer networks. The networks were studied with rheology and dynamic mechanical analysis. The mechanism of network rearrangement was studied using variable temperature ^31^P solid-state NMR. Hydrolytic- and thermal stabilities were tested and compared to the phosphate triester-based dynamic covalent networks with pendent β-hydroxy that we reported on previously. Finally, the catalytic properties of the β-hydroxy group on the transesterification reaction was studied.

## Results and discussion

**Fig. 1:**
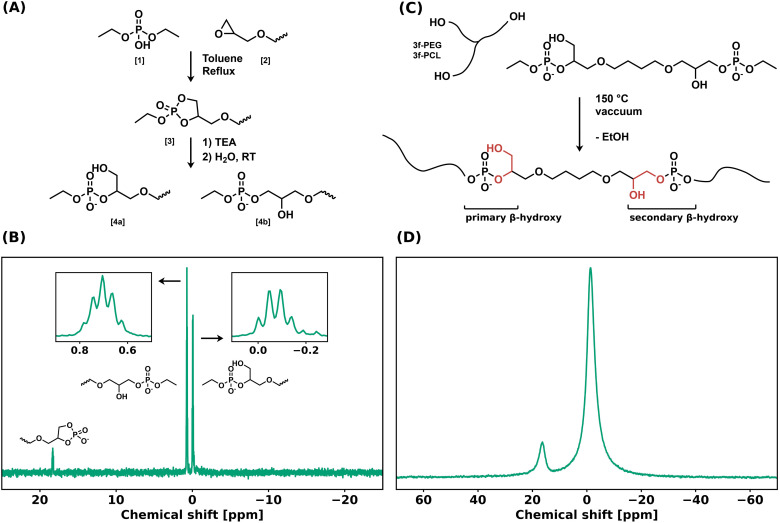
(a) Synthetic scheme for BDDE-dP-TEA from BDDE *via* BDDE-dcP. (b) ^31^P NMR spectrum of BDDE-dP-TEA. Insets are zoom-ins for the two major peaks. (c) Synthetic scheme for PEG-dP-TEA and PCL-dP-TEA networks from BDDE-dP-TEA. (d) ^31^P SSNMR spectrum of PEG-dP-TEA at room temperature.

A bifunctional linker (BDDE-dP-TEA) was synthesized in two steps from diethyl phosphate (DEP, 1) and 1,4-butanediol diglycidyl ether (BDDE, 2) (Fig. 1a). A biscyclic intermediate (BDDE-dcP, 3) was formed by refluxing BDDE and DEP in toluene for 18 hours. The BDDE-dP-TEA linker (4) was subsequently obtained by partial hydrolysis of BDDE-dcP in the presence of an organic base (triethylamine, TEA) and water. This results in a bifunctional linker with two anionic phosphate diesters with triethylammonium as counterion (p*K*_a_ = 10.75). In the final step, the 5-membered ring can open in two ways, yielding either a primary hydroxy group (4a) or a secondary hydroxy group (4b). ^31^P NMR (Fig. 1b) showed two distinct peaks belonging to the anionic phosphate diester at 0.70 ppm and −0.07 ppm, and a minor peak at 18.26 ppm, belonging to a cyclic byproduct. The major peaks were assigned to the corresponding structures based on their multiplicity.

The bifunctional linker BDDE-dP-TEA was used to synthesize polymer networks containing exclusively anionic phosphate diesters as dynamic moiety. Commercially available polycaprolactone-triol (PCL-3f, *M*_n_ = 1.93 kg mol^−1^) or polyethylene glycol-triol (PEG-3f, *M*_n_ = 1.0 kg mol^−1^) was reacted with BDDE-dP-TEA in an equimolar ratio to yield a polymeric network, PCL-dP-TEA or PEG-dP-TEA, respectively (Fig. 1c). Network formation was carried out at 150 °C under reduced pressure for 2 hours, after which the material was transferred to a vacuum oven at 180 °C overnight, to ensure further curing. The resulting networks were reprocessed by means of compression molding under 1 bar at 150 °C for 30 minutes and then slowly cooled under pressure to 100 °C followed by active cooling to room temperature. The gel fractions and swelling ratios of the reprocessed samples were determined using THF as solvent. PCL-dP-TEA had a gel fraction of 81% and a swelling ratio of 2.73; PEG-dP-TEA had a gel fraction of 82% and a swelling ratio of 1.42. The ^31^P solid-state NMR spectrum of PEG-dP-TEA at room temperature (Fig. 1d) showed two broad peaks at −1.41 ppm and 16.38 ppm, corresponding to the linear anionic phosphate diesters and the 5-membered cyclic anionic phosphate diesters, respectively.

To investigate potential effects of counterions in this system, PEG networks with more acidic counterions pyridinium (p*K*_a_ = 5.2) and methylimidazolium (p*K*_a_ = 6.95) were also prepared, yielding PEG-dP-Pyr and PEG-dP-MIM, respectively. It was found that these counterions do not notably affect network rearrangement kinetics (for details see the ESI†). This implies that these weakly acidic counterions (p*K*_a_ > 4) do not protonate the anionic phosphate diester (p*K*_a_ = 1.25) enough to contribute to the transesterification reaction. This is in line with what has been shown before in literature, where isomerization of anionic phosphate diesters was shown to be pH-independent at pH > 4.^41^ Furthermore, synthesis of networks with alkali metal counterions was considered, but was found to be non-trivial. It was therefore decided to solely continue with triethylammonium as counterion.

### Thermal- and rheological characterization

**Fig. 2:**
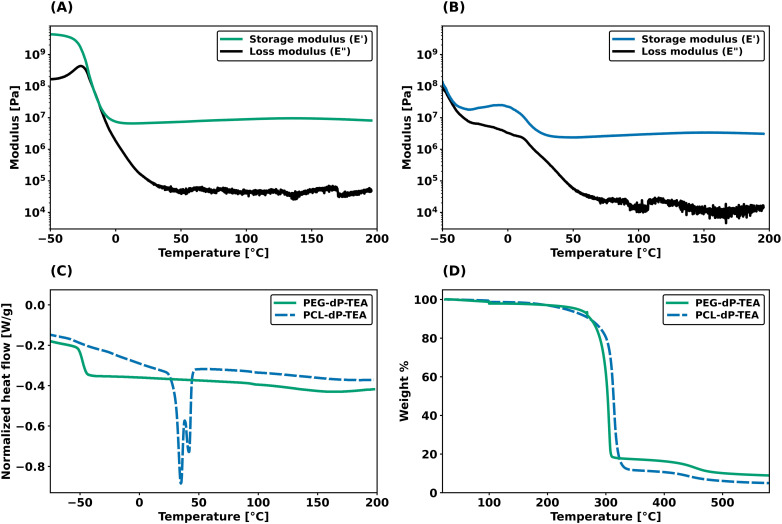
Thermal analysis of PEG-dP-TEA and PCL-dP-TEA. (a) DMTA profile of PEG-dP-TEA under 1 Hz oscillation. (b) DMTA profile of PCL-dP-TEA under 1 Hz oscillation. (c) DSC thermogram of the second heating run (10 °C min^−1^) of PEG-dP-TEA and PCL-dP-TEA (*exo* up). (d) TGA plot of PEG-dP-TEA and PCL-dP-TEA under N_2_ flow.

Dynamic mechanical thermal analysis (DMTA) was performed on the networks to explore the thermo-mechanical properties of the materials (Fig. 2a and b). DMTA of PEG-dP-TEA showed a drop in extensional storage modulus (*E*′) attributed to the glass transition temperature (*T*_g_) around −20 °C, followed by a rubbery plateau with a modulus of 10 MPa. The rubbery plateau shows a gradual increase with temperature until 140 °C, after which *E*′ gradually decreases. PCL-dP-TEA has a *T*_g_ at temperatures below −50 °C, followed by a modest increase in *E*′ at temperatures between −30 °C and −5 °C, representing cold crystallization. The material has a melting transition (*T*_m_) around 15 °C, followed by a rubbery plateau at 3 MPa. Again, the storage modulus of the material gradually increased with temperature, followed by a slight decrease above 150 °C. The DMTA profile of a generic polymer network with constant crosslink density shows a linear increase of storage modulus with temperature. In contrast, both PEG-dP-TEA and PCL-dP-TEA show a gradual decrease in *E*′ at elevated temperatures, indicative for a dissociative DCN due to the decrease in crosslink density at these temperatures caused by a shift in equilibrium towards the dissociated state. Differential scanning calorimetry (DSC, Fig. 2c) showed a *T*_g_ around −50 °C for both PEG-dP-TEA and PCL-dP-TEA. A *T*_m_ was visible for PCL-dP-TEA around 40 °C. Thermogravimetric analysis (TGA) was used to assess the thermal stability of the networks (Fig. 2d). The materials showed less than 5% weight loss until 250 °C for PEG-dP-TEA and 230 °C for PCL-dP-TEA.

**Fig. 3:**
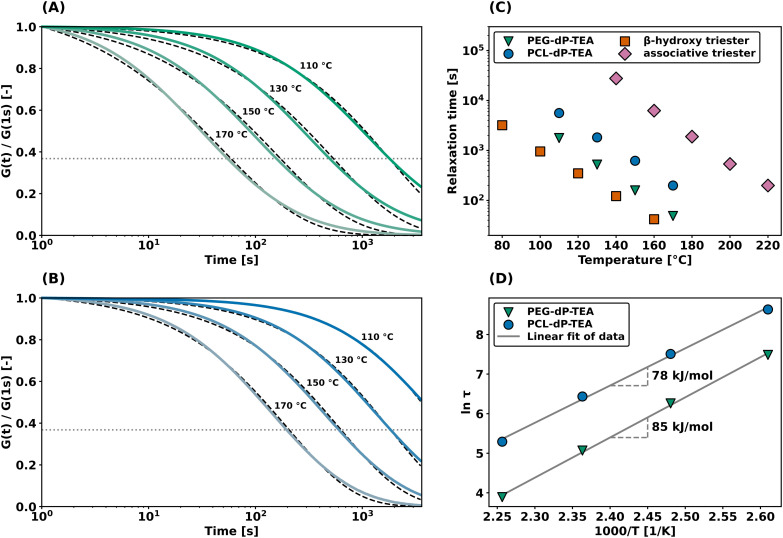
Shear rheology experiments under 1% strain with temperature variation. (a) Stress relaxation experiment on PEG-dP-TEA including fitted data (dashed lines). (b) Stress relaxation experiment on PCL-dP-TEA including fitted data (dashed lines). (c) Characteristic relaxation times of PEG-dP-TEA, PCL-dP-TEA, a β-hydroxy containing phosphate triester DCN^33^ and an associative phosphate triester DCN,^16^ (d) Arrhenius plot using characteristic relaxation times derived from (a) and (b). Values in the graph are the apparent activation energies of flow as calculated from the linear fit.

Additionally, shear rheology data was obtained to probe network rearrangement kinetics in PEG-dP-TEA and PCL-dP-TEA. Stress relaxation experiments were performed between 110 °C and 170 °C under 5% strain, results of which are shown as normalized curves in Fig. 3a and b (DMTA results show the modulus to not significantly change in this range of temperatures). Both networks exhibit full stress relaxation at elevated temperatures, with increasing relaxation rates as temperature increased. At 170 °C, complete stress relaxation was achieved within 2000 seconds for PEG-dP-TEA, and 3500 seconds for PCL-dP-TEA. The characteristic relaxation times (*τ*) were estimated by fitting the relaxation moduli, *G*(*t*), to a stretched exponential function, with stretch parameter *β* (eqn (1)).1
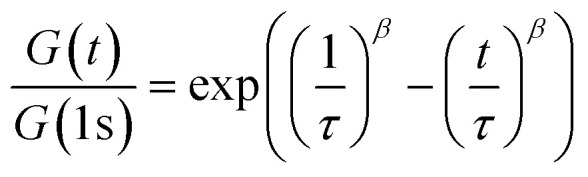


As shown in Fig. 3c, relaxation times for the β-hydroxy diester networks are generally shorter than for associative phosphate triester DCNs,^16^ yet higher than for the dissociative β-hydroxy containing triesters.^33^ This lies within expectation, due to the lower reactivity towards nucleophilic substitution of anionic phosphate diesters compared to phosphate triesters. An Arrhenius plot of the temperature dependence of fitted relaxation times (Fig. 3d) shows the apparent activation energy of viscous flow (*E*_a_) to be 85 kJ mol^−1^ for PEG-dP-TEA and 78 kJ mol^−1^ for PCL-dP-TEA, both marginally higher than our previously reported value of 68 kJ mol^−1^ for β-hydroxy containing phosphate triesters.^33^ The higher relaxation rates for PEG-dP-TEA compared to PCL-dP-TEA are attributed to the more polar backbone of PEG, stabilizing the transition states of the substitution.^42^

**Fig. 4:**
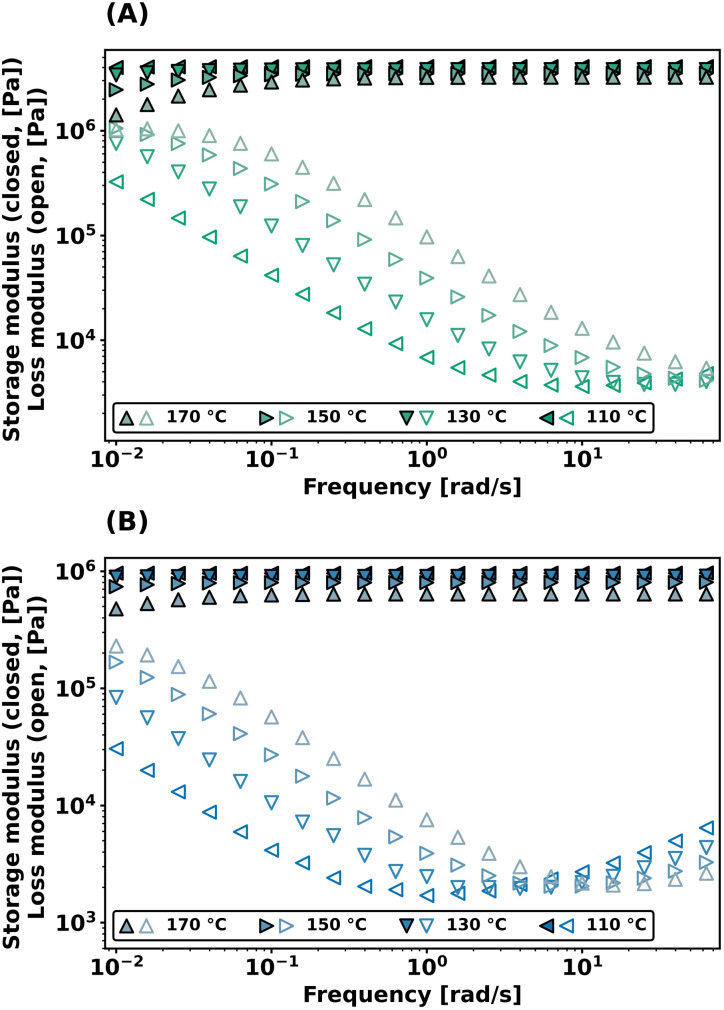
Frequency sweep data at different temperatures under 1% strain of (a) PEG-dP-TEA and (b) PCL-dP-TEA.

Dynamic bond exchange effects on the viscoelastic properties of the networks were further probed with frequency sweep experiments in shear, conducted from 10^−2^ to 10^2^ rad s^−1^ at different temperatures. As shown in Fig. 4, both PEG-dP-TEA and PCL-dP-TEA show a constant plateau modulus at higher frequencies (>0.5 rad s^−1^) which drops gradually with higher temperatures. Towards lower frequencies, the storage modulus (*G*′) decreases, accompanied by an increase in the loss modulus (*G*′′), indicating relaxation from the bond exchange reaction. The onset in drop of *G*′ shifts to higher frequencies with increasing temperature, and the plateau modulus decreases with increasing temperature, which is indicative for a dissociative exchange mechanism.^2^

### Rearrangement mechanism

**Fig. 5:**
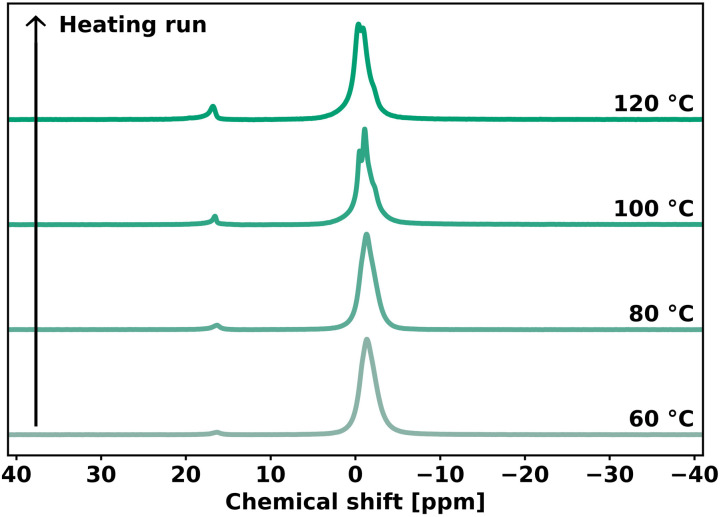
Heating run of VT ^31^P SSNMR measurement on PEG-dP-TEA.

The understanding of network rearrangement mechanisms is critical to the design of dynamic materials with tailored mechanical properties. The exchange mechanism in phosphate diesters has, to our knowledge, not yet been studied in the context of dynamic covalent networks. Previous studies assumed these moieties to show associative network rearrangement, but did not investigate this further.^37,38^ Variable temperature ^31^P solid-state NMR (VT ^31^P SSNMR) experiments were conducted to verify the dissociative network rearrangement as indicated by our rheology experiments. PEG-dP-TEA was allowed to equilibrate in the spectrometer at 60 °C, after which the temperature was increased to 120 °C with intervals of 20 °C. After 15 minutes of equilibration, a spectrum was acquired. As shown in Fig. 5, the VT ^31^P SSNMR spectrum of the heating run revealed an increase in concentration of 5-membered cyclic anionic phosphate diester, with the peak at 16.30 ppm becoming more intense with increasing temperature. In the cooling run (Fig. S28†), this peak partially diminishes, indicating the reversibility of the formation of the cyclic intermediate. This indicates a dissociative network rearrangement mechanism, where the increased concentration of the cyclic anionic phosphate diester at high temperatures shifts the equilibrium towards the dissociated state, as confirmed by the decrease in the rubbery plateau modulus with increasing temperature observed in the DMTA data. Furthermore, due to increased mobility in the sample at higher temperatures, the broad peaks attained a better resolution, revealing the peak assigned to the associated (ring-opened) state to consist of two peaks for the two positional isomers of the phosphate diester (Fig. 1c), and a downfield shoulder, that should probably be assigned to higher phosphates formed due to self-condensation reactions of the BDDE-dP-TEA linker.

The VT ^31^P SSNMR data from the heating run were used to evaluate the standard enthalpy for dissociation in the bulk polymer (Δ*H*^θ^). The ratio between the concentration of ring-opened state ([OP]) and 5-membered cyclic ring-closed ([CP]) was calculated from the VT ^31^P SSNMR peak integrals. For simplification, the peaks assigned to the two isomeric forms of the ring opened state were not evaluated separately, and a possible difference in reactivity between the primary- and secondary β-hydroxy was neglected. The concentration ratio ([CP]/[OP]) was used to evaluate Δ*H*^θ^ for dissociation using a Van ‘t Hoff plot (Fig. S29†), and was determined to be 26 kJ mol^−1^. This is slightly lower than the previously reported value of 35 kJ mol^−1^ for phosphate triesters,^33^ indicating that the cyclic phosphate diester intermediate is somewhat more stabilized than the cyclic triester intermediate.

### Material stability

**Fig. 6:**
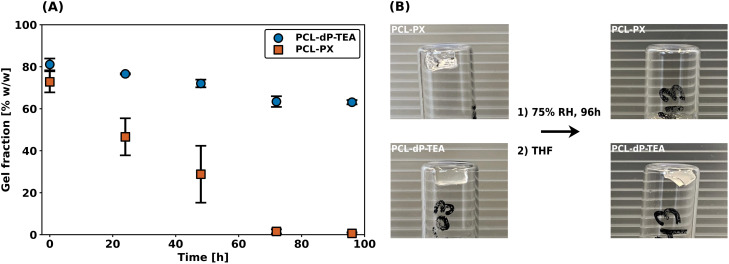
Degradation experiment of PCL-dP-TEA and PCL-PX. (a) Measured gel fraction over time. (b) Pictures after 0 h and 96 h of (top) PCL-PX and (bottom) PCL-dP-TEA.

As described in the introduction, the main reason to pursue anionic phosphate diesters as reactive moieties in DCNs is to improve hydrolytic- and thermal stabilities as compared to their triester counterparts. To investigate the hydrolytic stabilities of PCL-dP-TEA (based on diesters) and PCL-PX (based on triesters and synthesized as part of the work in ref. 33), degradation experiments were performed by exposing both networks to a 75% relative humidity atmosphere and determining the gel fraction (using THF as an extraction solvent) as a function of time. The results of these studies are shown in Fig. 6a and it is immediately clear that the PCL-PX network completely degraded within 4 days; this is also clearly shown in the pictures of Fig. 6b, where no network is visible anymore. In contrast, PCL-dP-TEA retained a significant gel fraction and maintained network integrity. These results provide a strong indication of the superior hydrolytic stability in the anionic phosphate diester-containing network as compared to phosphate triester.

**Fig. 7:**
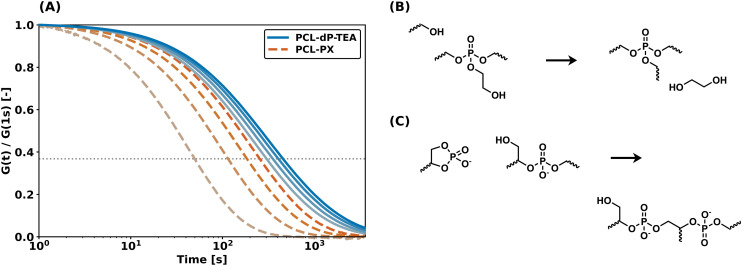
(a) Four cycles of shear stress relaxation experiments under 1% strain at 160 °C on single samples of PCL-dP-TEA and PCL-PX. (b) Reaction scheme of distant hydroxy-mediated expulsion of internal catalyst in PCL-PX. (c) Reaction scheme of formation of higher phosphates in PCL-dP-TEA. Note that here, the right phosphate centre loses its internal catalytic group.

We previously showed that the thermal stability in our triester-based network was limited because of the elimination of ethylene glycol through (associative) transesterification reactions at higher temperatures, thus eliminating the functional group responsible for the internal catalysis (Fig. 7b).^33^ This resulted in a significant increase in relaxation times in repeated stress relaxation experiments above 140 °C. Since the internal catalyst is incorporated in the linker (and thus the polymer backbone) in the diester-based material, this problem should have been eliminated in the current system. After confirming persistent stress relaxation in PCL-dP-TEA for temperatures up to 140 °C (see Fig. S17–S19†), the thermal stability of PCL-dP-TEA was compared to that of (previously synthesized)^33^ PCL-PX by means of repeated stress relaxation experiments at 160 °C on single samples (Fig. 7a). The differences in behavior for the two materials is immediately clear. For PCL-PX, the relaxation time over four cycles increased by 400%, whereas for PCL-dP-TEA, this was only 50%. We attribute the slight increase in relaxation time observed for PCL-dP-TEA to the formation of higher phosphates (Fig. 7c), as also shown using VT ^31^P SSNMR (Fig. 5).

### Influence of β-hydroxy group

The enhanced thermal- and hydrolytic stability of dissociative β-hydroxy diester networks compared to β-hydroxy triester networks is attributed to the less reactive nature of anionic phosphate diesters compared to phosphate triesters. Nonetheless, relatively fast stress relaxation is observed, hence a study on the catalytic influences of the present β-hydroxy group on the exchange reaction was conducted.

**Fig. 8:**
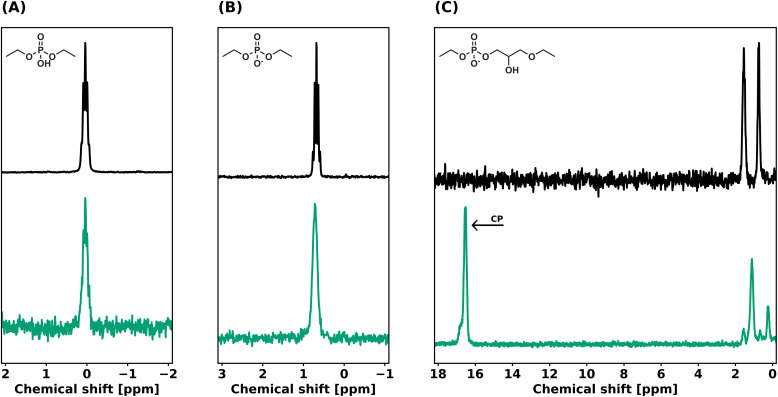
^31^P NMR of (A) (top) DEP, (bottom) reaction mixture of toluene, DEP, and 2-ethyl hexanol after reflux for 20 h. (B) (top) [EMIM][DEP], (bottom) reaction mixture of toluene, [EMIM][DEP], and 2-ethyl hexanol after reflux for 20 h. (C) (top) EGE-dP-TEA, (bottom) reaction mixture of toluene, EGE-dP-TEA, and 2-ethyl hexanol after reflux for 20 h. The arrow depicts the newly formed ring-closed state.

The influence of the β-hydroxy neighboring group as internal catalyst for network rearrangement was investigated through small molecule experiments. 3-Ethoxy-2-hydroxypropyl ethyl phosphate (EGE-dP) was synthesized from ethyl glycidyl ether (EGE) as a model for the dynamic moiety in the networks (Scheme S3†). Diethyl phosphate (DEP) and ethyl methyl imidazolium diethyl phosphate ([EMIM][DEP]) were used as a model for a neutral and anionic phosphate diester, respectively. EGE-dP, DEP and [EMIM][DEP] were refluxed in toluene for 20 h in the presence of 2-ethyl hexanol. From the ^31^P NMR spectra (Fig. 8) and the ^1^H NMR spectra (Fig. S5 and 6†) was found that no exchange occurred on the phosphate center in the case of DEP and [EMIM][DEP]. However, with EGE-dP, it was clear that exchange with 2-ethyl hexanol had occurred, as evidenced by the appearance of new peaks downfield in ^31^P NMR, assigned to exchanged centra (1.10 ppm and 0.24 ppm), and a peak assigned to the ring-closed state (16.5 ppm). These results suggest that the presence of a neighboring β-hydroxy group is essential for network rearrangement in an anionic phosphate diester-based DCN.

## Conclusions

In this work, we reported a dynamic covalent network based solely on anionic phosphate diesters as dynamic moieties. By synthesizing a bifunctional linker and combining this with commercially available polymeric triols, we obtained two networks with PEG (PEG-dP-TEA) and PCL (PCL-dP-TEA) as polymer backbones. Network rearrangements in these networks was activated at elevated temperatures and we demonstrated that rearrangement occurs *via* a dissociative mechanism, as evidenced by rheology and VT ^31^P SSNMR. The dissociative rearrangement goes *via* a 5-membered cyclic intermediate, similar to the mechanism as known from RNA self-cleavage.

The synthesized networks showed full and relatively fast stress relaxation at elevated temperatures, with PEG-dP-TEA showing notably higher relaxation rates than PCL-dP-TEA. We attributed this difference to the more polar PEG backbone providing more stability to the transition state. Although these networks show lower relaxation rates than our previous β-hydroxy containing phosphate triester network (PCL-PX),^33^ we showed a considerable increase in thermal- and hydrolytic stability. This shows that switching from phosphate triesters to anionic phosphate diesters can greatly improve material stability over time, by only slightly compromising rearrangement kinetics.

The catalytic effects of both the present β-hydroxy group and the counterion of the diester anion were investigated. Our results indicate that the counterion has negligible effect on the rearrangement kinetics, while the presence of the β-hydroxy group was shown to be vital for exchange to occur. This once more highlights the importance of neighboring group participation in (dissociative) dynamic covalent networks.

## Author contributions

The manuscript was written through contributions of all authors. All authors have given approval to the final version of the manuscript.

## Conflicts of interest

There are no conflicts to declare.

## Supplementary Material

PY-014-D3PY00867C-s001
