# Supplementary material for: RNA-inspired phosphate diester dynamic covalent networks
Source: Polym Chem. 2023 Sep 1;14(37):4294–302. doi: 10.1039/d3py00867c (PMC10520920; doi:10.1039/d3py00867c)
Supplement: PY-014-D3PY00867C-s001 [file PY-014-D3PY00867C-s001.pdf]

Electronic Supplementary Material (ESI) for Polymer Chemistry.
This journal is © The Royal Society of Chemistry 2023

Supporting information

# RNA-Inspired Phosphate Diester Dynamic Covalent Networks

Roy Wink[1], Soumabrata Majumdar[1], Rolf A.T.M. van Benthem[2],
Johan P.A. Heuts[1*], Rint P. Sijbesma[1*]

1: Department of Chemical Engineering & Chemistry, and Institute for Complex Molecular Systems, Eindhoven University of Technology, P.O. Box 513, 5600 MB Eindhoven (The Netherlands).

2: Department of Chemical Engineering & Chemistry, Laboratory of Physical Chemistry, Eindhoven University of Technology, P.O. Box 513, 5600 MB Eindhoven (The Netherlands) and Shell Energy Transition Center Amsterdam, Grasweg 31, 1031 HW Amsterdam (The Netherlands).

*Corresponding authors:

j.p.a.heuts@tue.nl
r.p.sijbesma@tue.nl

# Contents

Materials ... 3

Syntheses ... 3

Synthesis of diethyl phosphate (DEP) ... 3

Synthesis of BDDE-dP-TEA via BDDE-dcP ... 5

Network synthesis ... 6

Reprocessing via compression molding ... 6

Small molecule experiments for catalysis study ... 6

Characterization ... 9

Gel fraction ... 9

Swelling ratio ... 9

Nuclear magnetic resonance (NMR) spectroscopy ... 9

(Variable temperature) solid-state NMR (SSNMR) spectroscopy ... 10

Determination of $\Delta H^{\Theta}$ ... 10

Thermogravimetric analysis (TGA) ... 10

Differential scanning calorimetry (DSC) ... 11

Dynamic mechanical thermal analysis (DMTA) ... 11

Shear rheology ... 11

Oscillatory time sweep experiments ... 11

Stress relaxation experiments ... 11

Repeated stress relaxation experiments for thermal stability ... 11

Frequency sweep experiments ... 12

Relaxation modes via Tikhonov regularization ... 12

Degradation experiments ... 12

Supporting results ... 13

BDDE-dP-TEA NMR spectra ... 13

Stress relaxation experiments ... 15

Normalized data and Arrhenius fit ... 15

Relaxation modes via Tikhonov regularization ... 18

Repeated stress relaxation experiments ... 19

Dynamic mechanical thermal analysis ... 21

Differential scanning calorimetry ... 23

VT $^{31}P$ SSNMR ... 25

Supporting references ... 26

# Materials

1-Ethyl-3-methylimidazolium diethyl phosphate >96% (**[EMIM][DEP]**), ethyl glycidyl ether >98% (**EGE**), and 2-ethyl hexanol >99.5% (**2-EH**) were obtained from TCI Europe. 1,4-butanediol diglycidyl ether ≥95% (**BDDE**), triethylamine ≥99.5% (**TEA**), 1-methylimidazole 99% (**MIM**), glycerol ethoxylate (**PEG-3f**) and Amberlite® IRC120 hydrogen form were obtained from Merck. Toluene AR, tetrahydrofuran (stab./BHT) extra dry (**THF**) and pyridine AR were obtained from Biosolve. CAPA® 3201; a caprolactone/trimethylolpropane copolymer (**PCL-3f**) was kindly provided by Ingevity. All compounds were used as-is, without further drying, unless stated otherwise.

A phosphate triester-containing polycaprolactone network (**PCL-PX**) was synthesized from CAPA® 3201 and ethylene glycol chlorophosphate as described by our group before.[1]

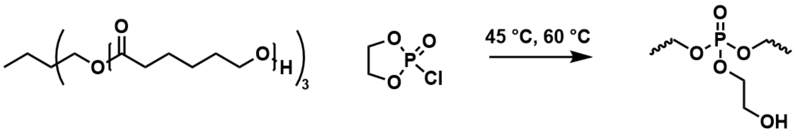


*Scheme S1: Synthesis of **PCL-PX***

# Syntheses

## Synthesis of diethyl phosphate (DEP)

**DEP** (**1**) was made from **[EMIM][DEP]** as described before in the literature.[2]

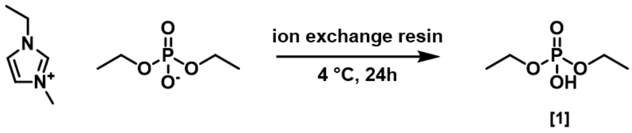


*Scheme S2: Synthesis of diethyl phosphate*

80-90 g thoroughly washed Amberlite® IRC120 hydrogen form and 150 mL water was added to a 250 mL Erlenmeyer flask together with 10 g 1-ethyl-3-methylimidazolium diethyl phosphate (**[EMIM] [DEP]**). This was left in the fridge for 24 hours. The mixture was filtered through filter paper and washed with fresh water into a 250 mL round bottom flask. Water was removed from the filtrate by means of reduced pressure, followed by threefold co-evaporation with toluene. This yielded protonated diethyl phosphate (**DEP**) in quantitative conversion (5.78 g, 99%) in the form of a yellow liquid. In the $^{31}$P NMR spectrum (Figure S1), a shift is visible towards lower chemical shift, indicating the protonation.

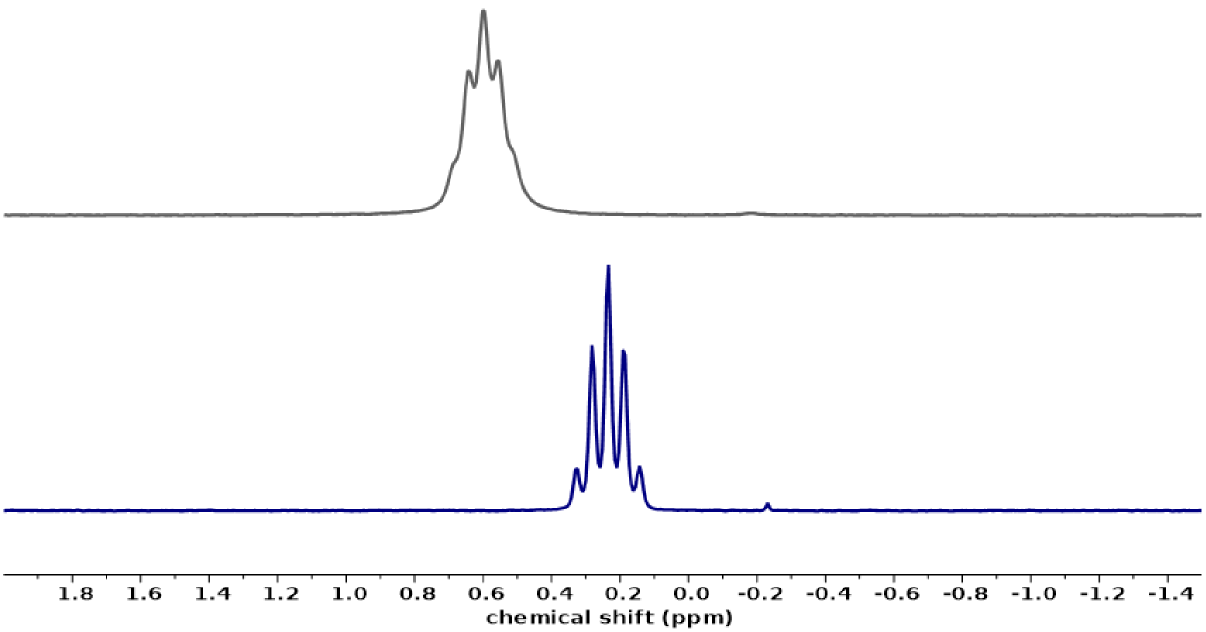


*Figure S1: $^{31}P$ NMR of (top)* ***[EMIM][DEP]*** *(162 MHz, $H_2O+D_2O$) δ 0.60 (t, J = 7.6 Hz). (bottom)* ***DEP*** *(162 MHz, $H_2O+D_2O$) δ 0.24 (p, J = 7.6 Hz).*

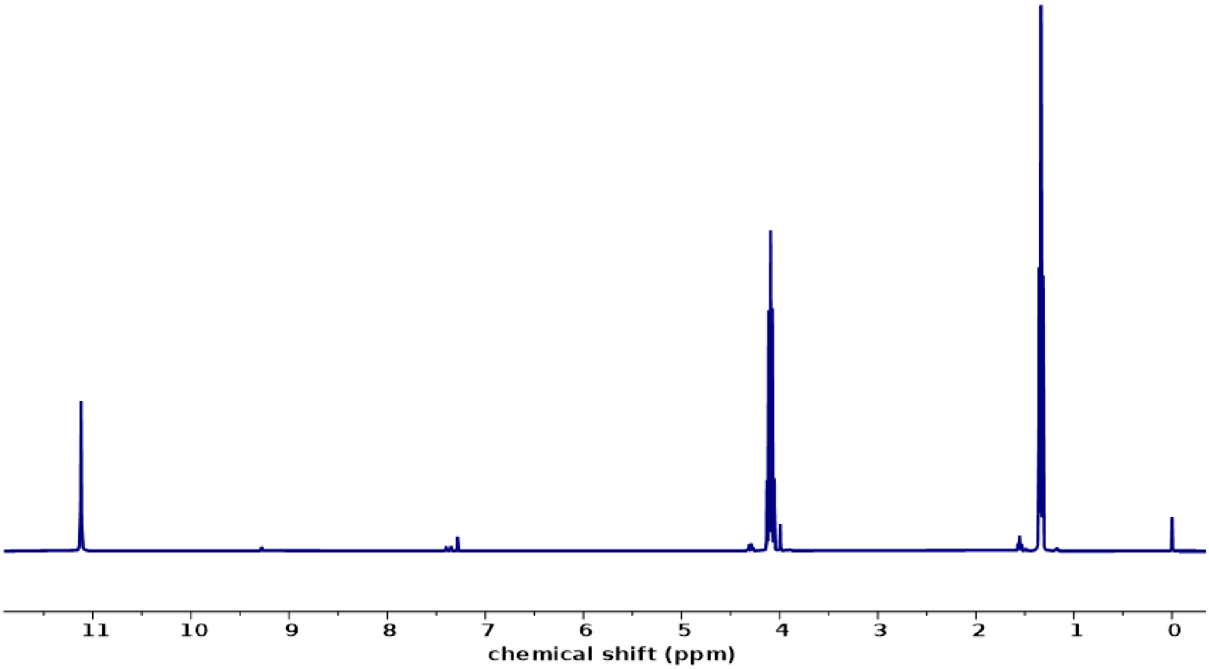


*Figure S2: $^{1}H$ NMR of* ***DEP*** *(400 MHz, $CDCl_3$) δ 11.12 (s, 1H, P-**OH**), 4.14 – 4.04 (m, 4H, P-O-**$CH_2$**-$CH_3$), 1.34 (t, J = 7.1, 1.0 Hz, 6H, P-O-$CH_2$-**$CH_3$**).*

## Synthesis of BDDE-dP-TEA via BDDE-dcP

**BDDE-dcP** (**3**) was made from **BDDE** (**2**) and **DEP** (**1**) as described in the literature.[3] **BDDE-dcP** was then partially hydrolyzed into **BDDE-dP-TEA** (**4**).

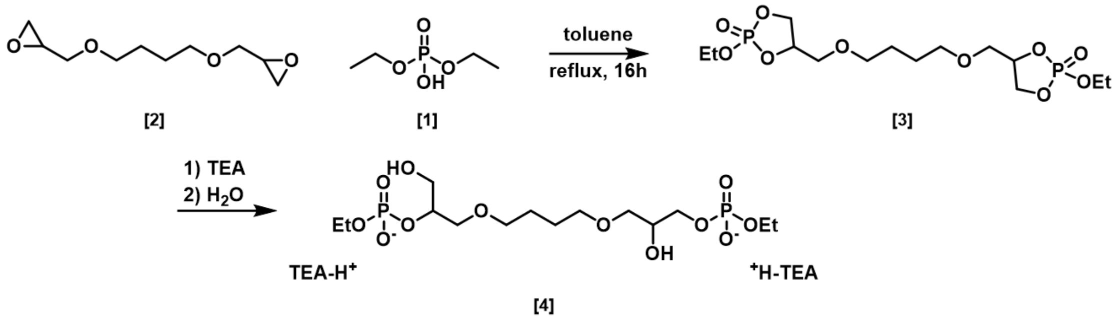


*Scheme S3: Synthesis of **BDDE-dcP** and **BDDE-dP-TEA***

2 g **DEP** (13.0 mmol) was dissolved in 400 mL dry toluene that was dried over 4 Å molecular sieves, together with an equimolar amount (6.5 mmol, 1.31 g) 1,4-butanediol diglycidyl ether (**BDDE**). A 20 mL Dean-Stark apparatus was attached, of which the burette was filled with 4 Å molecular sieves. The mixture was refluxed overnight. The reaction mixture was cooled down and filtered into a 500 mL separation funnel to filter off orange solid side-products. Only one peak was visible in the $^{31}$P NMR spectrum (Figure S3 top), which was attributed to the ring-closed **BDDE-dcP**. 20 mL Triethylamine (TEA) was added and the mixture was mixed well. 25 mL water was added, the mixture was shaken multiple times, and left to equilibrate. The aqueous layer was added to a 100 mL round bottom flask and the organic layer was washed with water two more times before being discarded. The combined aqueous layers were freed from water by means of reduced pressure to yield **BDDE-dP-TEA** as a colorless to slightly yellow ionic liquid in 72% conversion (3.24 g). From $^{31}$P NMR, (Figure S3 bottom), it was visible that the two positional isomers of the first hydrolysis product are formed. $^{1}$H, $^{13}$C, and $^{31}$P NMR of **BDDE-dP-TEA** are shown below in section *BDDE-dP-TEA NMR spectra*.

Replacing **TEA** in this synthetic approach for pyridine (**Pyr**) or methyl imidazole (**MIM**) yields **BDDE-dP-Pyr** or **BDDE-dP-MIM**, respectively. For **MIM**, the aqueous layer after hydrolysis was washed with toluene for a total of 8 times to remove all unreacted **MIM**.

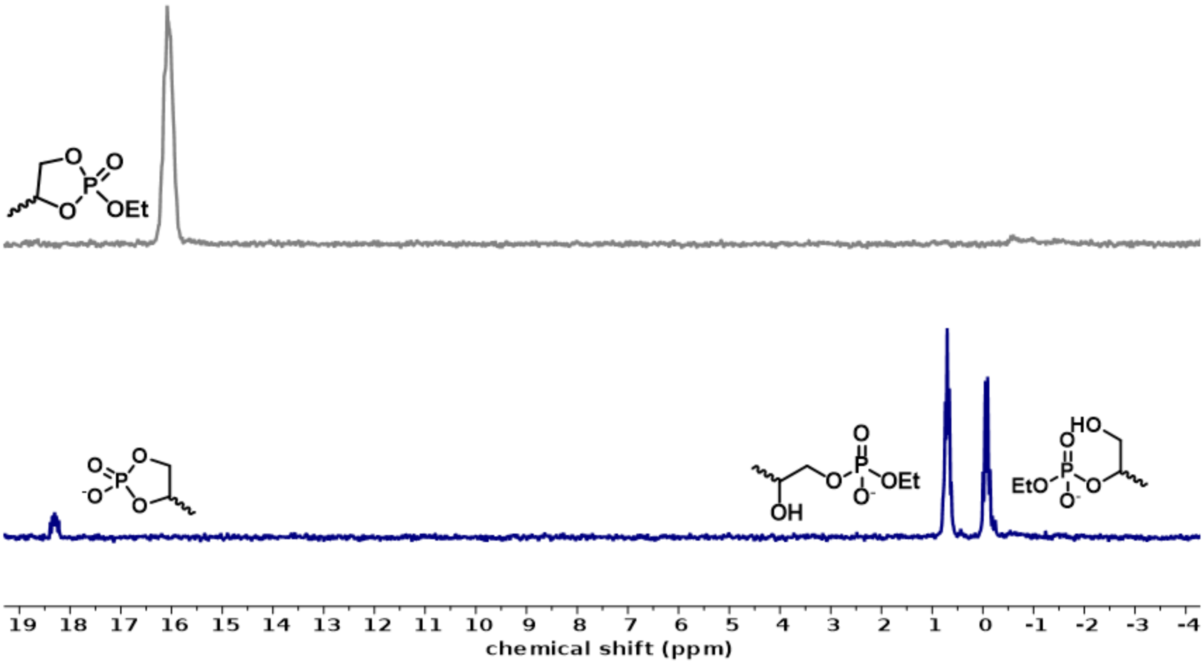


*Figure S3: $^{31}$P NMR of (top)* ***BDDE-dcP*** *(162 MHz, Toluene (no D)) δ 16.03 (m). (bottom)* ***BDDE-dP-TEA*** *(162 MHz, $H_2O$+$D_2O$) δ 0.64 (p, J = 6.3 Hz), -0.12 (q, J = 6.8 Hz).*

## Network synthesis

For network synthesis, an equimolar amount (with respect to end groups) of **BDDE-dP-TEA** and a three-functional, OH-terminated polymer (**PEG-3f**, **PCL-3f**) were combined in a 100 mL round bottom flask. The mixture was heated to 60 °C and stirred to combine the fractions. Next, the mixture was freed from any residual water by means of lyophilization in case of **PEG**, and by means of threefold coevaporation with toluene in case of **PCL**. Afterwards, the mixture was stirred well, and under high vacuum (< 1 mbar) the temperature was increased to 150 °C until network formation was complete. Vacuum was necessary to remove ethanol. Afterwards, the material was collected in a glass petri dish and left in a vacuum oven at 180 °C to ensure further curing

## Reprocessing via compression molding

Synthesized networks were pressed into 1 - 1.2 mm thick squares, from which 8 mm disk were punched for rheology experiments, and rectangles were cut for DMTA experiments. For all other characterization methods, pressed scraps were used. Compression molding was performed in a Fontijne LabEcon 300 hot press using a set method. Material was placed in a mold and sandwiched between 0.05 mm thick Teflon sheets and 4 mm thick stainless steel sheets. This was placed under 100 kN at 150 °C for 30 minutes. The temperature was reduced to 100 °C over the course of 30 minutes (under pressure) and then the samples were actively cooled to room temperature.

## Small molecule experiments for catalysis study

**EGE** (**5**) was phosphorylised into **EGE-dP-TEA** (**7**) in a comparable manner to **BDDE**. 2 g **DEP** (13.0 mmol) was dissolved in 300 mL toluene that was dried over 4 Å molecular sieves, together with an equimolar amount (13.0 mmol, 1.4 mL) **EGE**. A 20 mL Dean-Stark apparatus was attached, of which the burette was filled with 4 Å molecular sieves. The mixture was refluxed overnight. The reaction mixture was cooled down and filtered into a 500 mL separation funnel to filter off solid side-products.

One major peak was visible in the $^{31}P$ NMR spectrum (Figure S4 top), which was attributed to the ring-closed **EGE-dcP** (**6**), as well as one minor peak, which was attributed to an unknown side product. 20 mL triethylamine (**TEA**) was added and the mixture was mixed well. 25 mL water was added, the mixture was mixed multiple times, and left to equilibrate. The aqueous layer was added to a 100 mL round bottom flask and the organic layer was washed with water two more times before being discarded. The combined aqueous layers were dried by means of reduced pressure to obtain **EGE-dP-TEA** as a colorless ionic liquid in 47% conversion (2.00 g). From $^{31}P$ NMR (Figure S4 bottom), it was visible that the two positional isomers of the first hydrolysis product are formed.

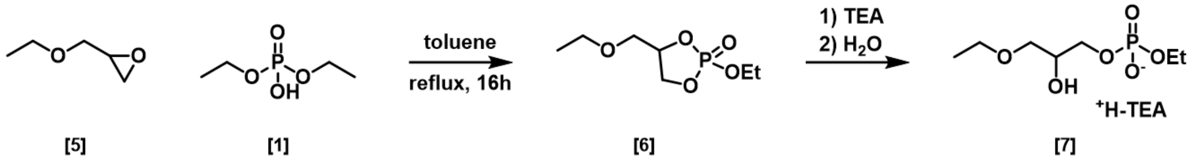


*Scheme S4: Synthesis of **EGE-dP-TEA** via **EGE-dcP**.*

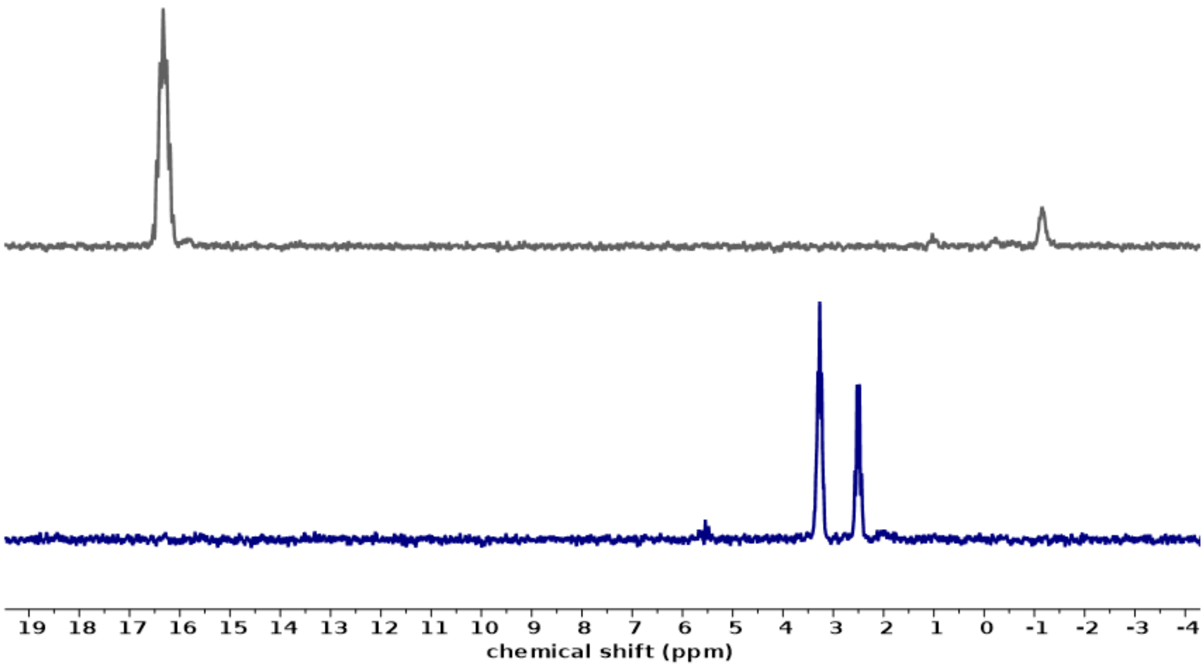


*Figure S4: $^{31}P$ NMR of (top) **EGE-dcP** (162 MHz, Toluene (no D)) δ 16.33 (m), -1.16 (s). (bottom) **EGE-dP-TEA** (162 MHz, $H_2O$+$D_2O$) δ 3.27 (p, J = 6.8 Hz), 2.50 (q, J = 7.6 Hz).*

For small molecule exchange reactions, 1g **DEP**, **[EMIM][DEP]**, or **EGE-dP-TEA** was dissolved in 30 mL toluene that was dried over 4 Å molecular sieves, together with an equimolar amount 2-ethyl hexanol (1:2 for **DEP** and **[EMIM][DEP]**, 1:1 for **EGE-dP-TEA**). The mixture was refluxed for 20 hours.

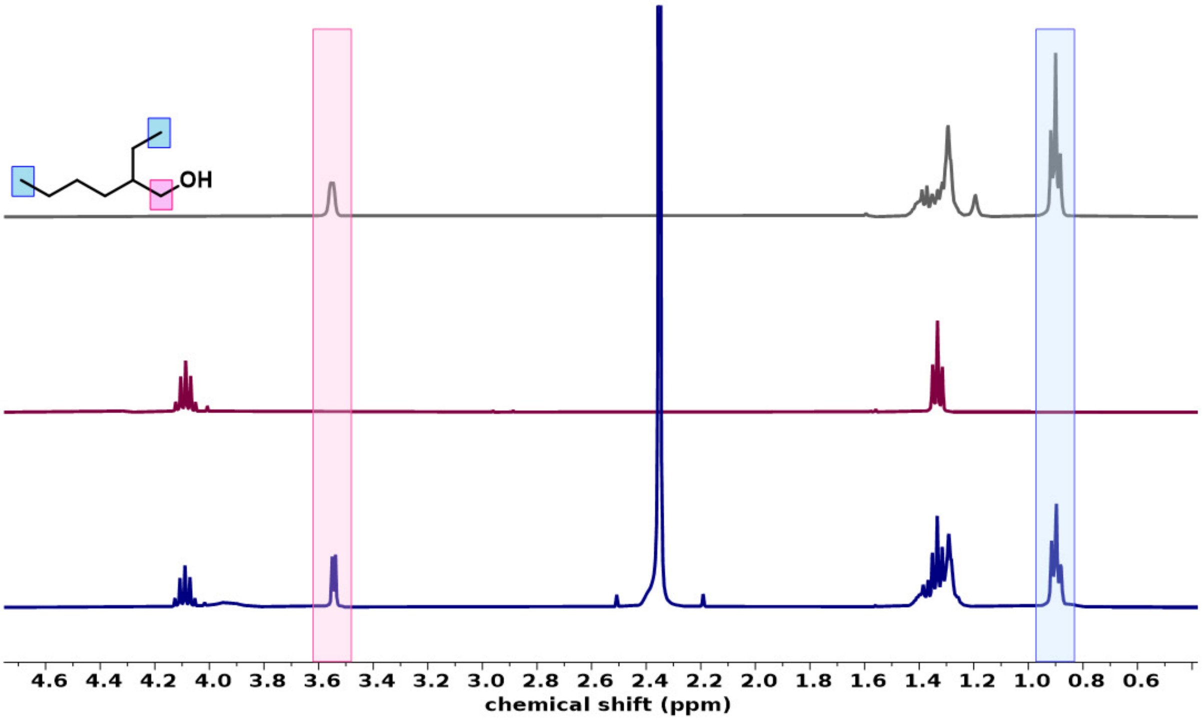


*Figure S5: $^{1}H$ NMR of (top) 2-ethyl hexanol, (middle)* ***DEP****, (bottom) reaction mixture of toluene,* ***DEP****, and 2-ethyl hexanol. From integration of the marked areas was determined that no exchange reaction occurred.*

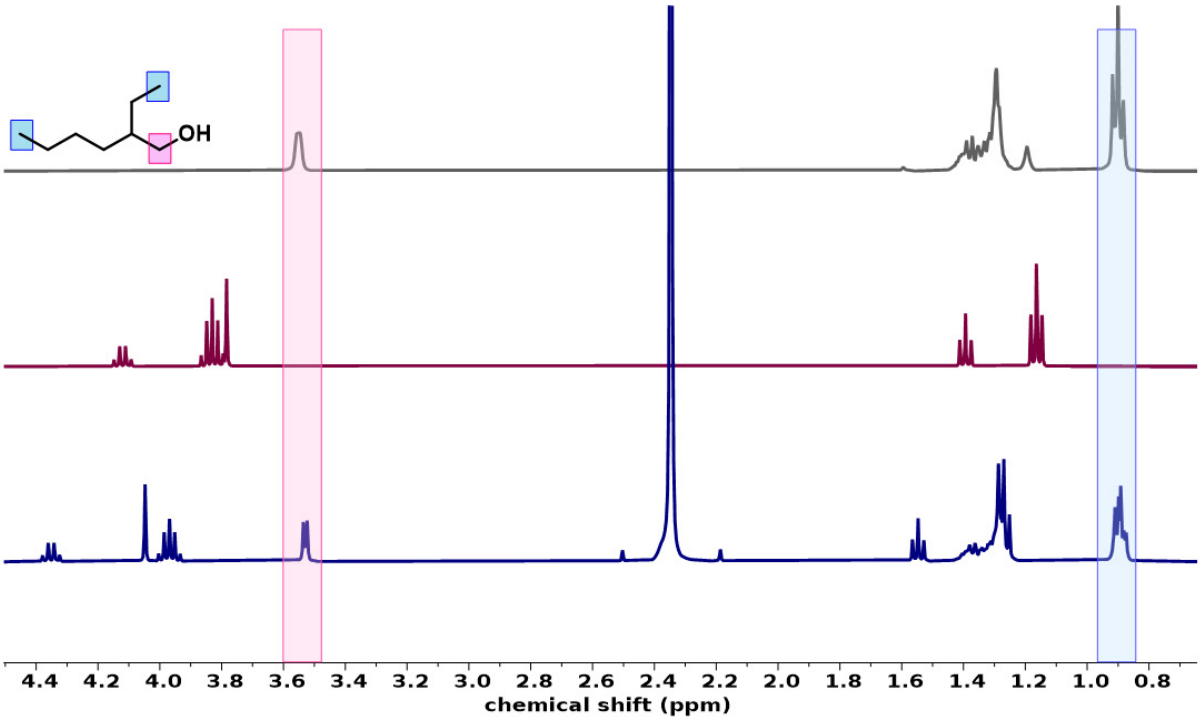


*Figure S6: $^{1}H$ NMR of (top) 2-ethyl hexanol, (middle)* ***[EMIM][DEP]****, (bottom) reaction mixture of toluene,* ***[EMIM][DEP]****, and 2-ethyl hexanol. From integration of the marked areas was determined that no exchange reaction occurred.*

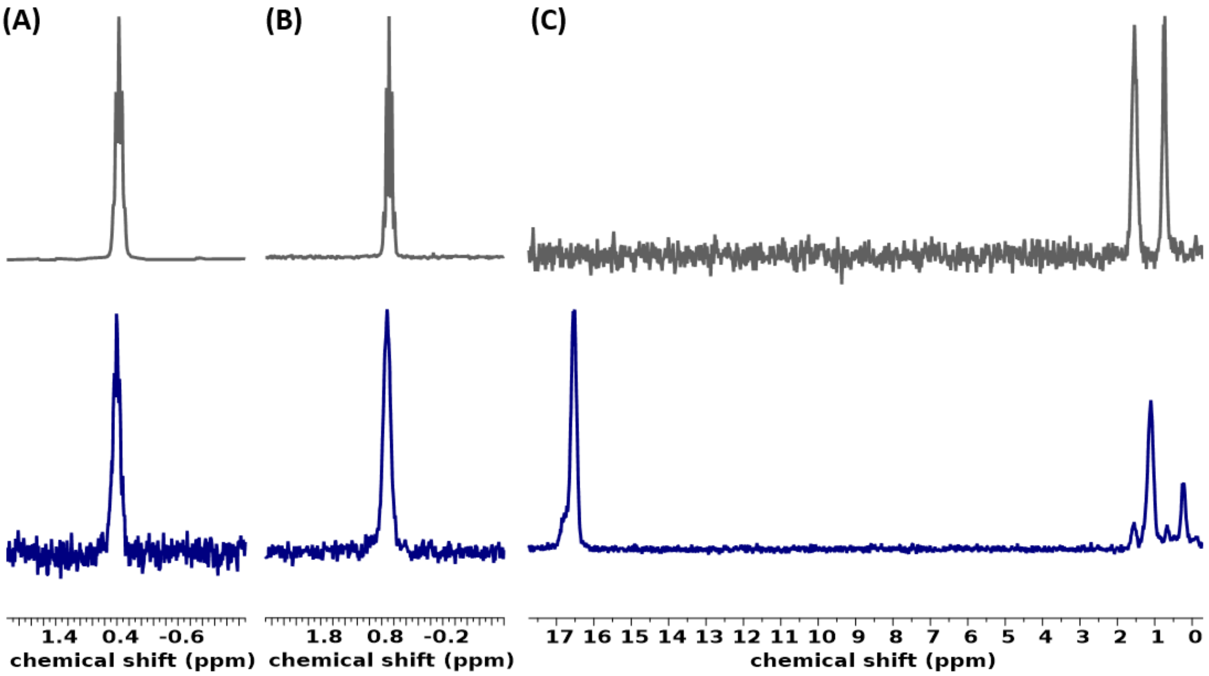


*Figure S7: $^{31}$P NMR of (A) (top)* ***DEP****, (bottom) reaction mixture of toluene,* ***DEP****, and 2-ethyl hexanol. (B) (top)* ***[EMIM][DEP]****, (bottom) reaction mixture of toluene,* ***[EMIM][DEP]****, and 2-ethyl hexanol. (C) (top)* ***EGE-dP-TEA****, (bottom) reaction mixture of toluene,* ***EGE-dP-TEA****, and 2-ethyl hexanol.*

# Characterization

## Gel fraction

Gel fractions of the networks were determined with tetrahydrofuran (**THF**) as the extraction solvent. A piece of (dry) sample was weighed ($m_i$) and then swollen in the extraction solvent for 24 hours. The swollen network was then washed with fresh solvent and allowed to dry in a vacuum oven at 100 °C overnight. The dried sample was weighed ($m_d$) and the gel fraction was calculated following equation S1.

$$gel\ fraction\ (\%) = \frac{m_d}{m_i} \cdot 100\%$$ *Equation S1*

## Swelling ratio

Swelling ratio was determined using THF as solvent. A piece of (dry) sample was weighed ($m_i$) and allowed to swell in the solvent for 24 hours. The swollen sample was pat dry with a piece of paper and weighed ($m_s$). Swelling ratio ($w$) was calculated flowing equation S2.

$$w = \frac{m_s}{m_i}$$ *Equation S2*

## Nuclear magnetic resonance (NMR) spectroscopy

$^{1}$H, $^{13}$C and $^{31}$P NMR spectroscopy measurements were performed on a 400 MHz Bruker Avance III spectrometer at 25 °C.

## (Variable temperature) solid-state NMR (SSNMR) spectroscopy

VT $^{31}P$ SSNMR spectra were obtained using an 11.7 Tesla Bruker Avance Neo 500 MHz spectrometer operating at $^{31}P$ resonance frequency of 202 MHz. The measurements were performed under static conditions, without magic angle spinning, using a one pulse sequence with a 90° pulse of 5 µs and an inter-scan delay of 10 s. A 0.0485 M triphenylphosphate (TPhP) solution in $CDCl_3$ was used as a chemical shift reference at 17.70 ppm. The variable temperature experiment was performed from 60 °C to 120 °C at intervals of 20 °C up and down. The system was allowed to equilibrate until equilibrium was reached at 60 °C, as well as for 15 min at each temperature.

### *Determination of $\Delta H^{\Theta}$*

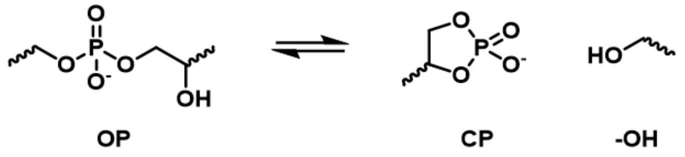


*Scheme S5: Equilibrium between the ring-opened and the ring-closed state of a phosphate diester DCN.*

Given the equilibrium as shown in Scheme S5, the equilibrium constant $K$ for the equilibrium between the ring-opened state and the ring-closed state in phosphate diester DCNs can be determined as represented in Equation S3.

$$K = \frac{[CP][-OH]}{[OP]} \qquad \textit{Equation S3}$$

Since 1:1 stoichiometry was used in the network synthesis, the networks will contain dissociated phosphate diesters and polymer -OH functionalities in a 1:1 ratio. All processes involved are transesterification reactions, hence the OH content in the network remains constant. The concentration ratio between the dissociated and associated phosphate esters can be calculated from the SSNMR spectra, as the ratio between areas of the corresponding peaks. Using Gibbs' fundamental equation and the Van 't Hoff's law, an expression can be derived for the [CP]/[OP] dependence of $\Delta H^{\Theta}$ (Equation S4).

$$\Delta G^{\Theta} = \Delta H^{\Theta} - T\Delta S^{\Theta} = -RT\ln K$$
$$\ln K = \frac{-\Delta H^{\Theta}}{RT} + \frac{\Delta S^{\Theta}}{R}$$
$$\ln\left(\frac{[CP]}{[OP]}\right) = \frac{-\Delta H^{\Theta}}{RT} + \frac{\Delta S^{\Theta}}{R} - \ln[-OH] \qquad \textit{Equation S4}$$

## Thermogravimetric analysis (TGA)

Thermal stability of the networks were probed using a TGA 550 (TA instruments) under $N_2$ flow. Temperature ramps were performed by ramping from 20 °C to 100 °C at 20 °C per minute, allowing an isothermal at 100 °C for 30 minutes, followed by a ramp to 600 °C at 10 °C per minute. Temperature calibration was performed using the Curie points of high purity ferromagnetic standards.

## Differential scanning calorimetry (DSC)

DSC was performed in a Q2000 DSC (TA instruments). Measurements were performed using standard aluminum pans. The temperature range used was -80 °C until 200 °C. The material was cooled to the minimum temperature at 5 °C per minute. Heating runs were performed at 10 °C per minute.

## Dynamic mechanical thermal analysis (DMTA)

DMTA was performed on rectangular shaped compression molded samples using the film tension setup in a Discovery DMA 850 (TA instruments). Samples were heated to 180 °C and left to equilibrate for 2 hours. The sample was cooled at 1° C/min under oscillation to -50 °C and left to equilibrate for 1 hour. Then, the temperature ramp was performed at 3 °C/min from -50 °C to 200 °C. A preload force of 0.01 N and a force track of 125% was used. The storage- and loss modulus were recorded as a function of temperature.

## Shear rheology

All shear rheology studies were performed using a Discovery HR 20 (TA instruments) with environmental temperature control (ETC) setup and 8 mm parallel-plate geometry. All experiments were performed with an axial force range of 1 ± 0.1 N. In general, all samples were equilibrated at 180 °C for 2.5 hours before measurement (unless stated otherwise).

### *Oscillatory time sweep experiments*

Oscillatory time sweep experiments were performed before each stress relaxation experiments at the same temperature to ensure proper curing at the experiment temperature. The experiments were performed under 1% oscillatory strain and at an angular frequency of 10 rad/s.

### *Stress relaxation experiments*

Stress relaxation experiments were performed between 110 °C and 170 °C. The relaxation modulus $G(t)$ was followed as a function of step time under a constant strain of 5%. All plots in main text have been normalized to the relaxation modulus at 1 s ($G(1s)$), in order to compare the relaxation rates solely based on the kinetics of bond exchange.

Fits of the normalized stress relaxation plots were made following a stretched exponential function normalized to 1 s, as seen in Equation S5, yielding the characteristic relaxation times ($\tau$) and the stretch parameter ($\beta$). The obtained characteristic relaxation times were plotted against the reciprocal temperatures via an Arrhenius plot. Slopes from these plots can be used to determine the apparent activation energy of viscous flow for the system (Equation S6).

$$\frac{G(t)}{G(1s)} = \exp\left(\left(\frac{1}{\tau}\right)^{\beta} - \left(\frac{t}{\tau}\right)^{\beta}\right) \qquad \textit{Equation S5}$$

$$\ln\tau = \ln\tau_0 + \frac{E_a}{RT} \qquad \textit{Equation S6}$$

### *Repeated stress relaxation experiments for thermal stability*

Stress relaxation experiments were repeated on single samples at equal temperatures four times in order to assess the thermal stability of the material. The same method was used as described above.

### *Frequency sweep experiments*

Frequency sweep experiments from $10^{-2}$ rad/s to $10^{2}$ rad/s were performed on the networks between 110 °C and 170 °C. The oscillation amplitude was set to 1% strain. The storage modulus, loss modulus and tan delta were followed as a function of frequency.

### *Relaxation modes via Tikhonov regularization*

Stress relaxation spectra of **PEG-dP-TEA** and **PCL-dP-TEA** were checked for plurality in relaxation modes via Tikhonov regularization. The used method has been described before,[4] and is based on solving the stress relaxation function for the distribution function $\sigma(\tau)$ as described in:

$$\sigma(t) = \sigma_e + \int_0^{+\infty} \sigma(\tau) \exp\left(-\frac{t}{\tau}\right) d\tau \qquad \textit{Equation S7}$$

Here, $\sigma(t)$ is defined as gradual stress relaxation to the equilibrium stress $\sigma_e$. Assuming stress is fully relaxed in the measurements ($\sigma_e = 0$), the integral in Equation S7 can be written in the generic form of a Fredholm equation of the first kind:

$$g(s) = \int_a^b K(s,t)\, f(t)\, dt\,, \qquad a \le s \le b \qquad \textit{Equation S8}$$

Where $g(s)$ is the measured signal, $K(s,t)$ the kernel function $\exp\left(-\frac{t}{s}\right)$ where $s$ depicts $\tau$ between $0$ and +∞; and $f(t)$ the unknown solution to the integral which needs be solved for. This is an example of an ill-posed problem, which thus requires regularization. Here, it is chosen to utilize Hansen's algorithms in MATLAB to solve the problem.[5] These employ the L-curve criterion to determine the optimum regularization parameter $\lambda$, and Tikhonov regularization for the computation of the relaxation spectra.

## Degradation experiments

Samples of **PCL-dP-TEA** and **PCL-PX** (Majumdar et al.[1]) were weighed and left in open vials in a desiccator containing saturated NaCl solution, maintaining a humidity of 75% at room temperature. For 5 days, three samples per network were removed per day, and the gel content was determined as described before.

## Supporting results

### BDDE-dP-TEA NMR spectra

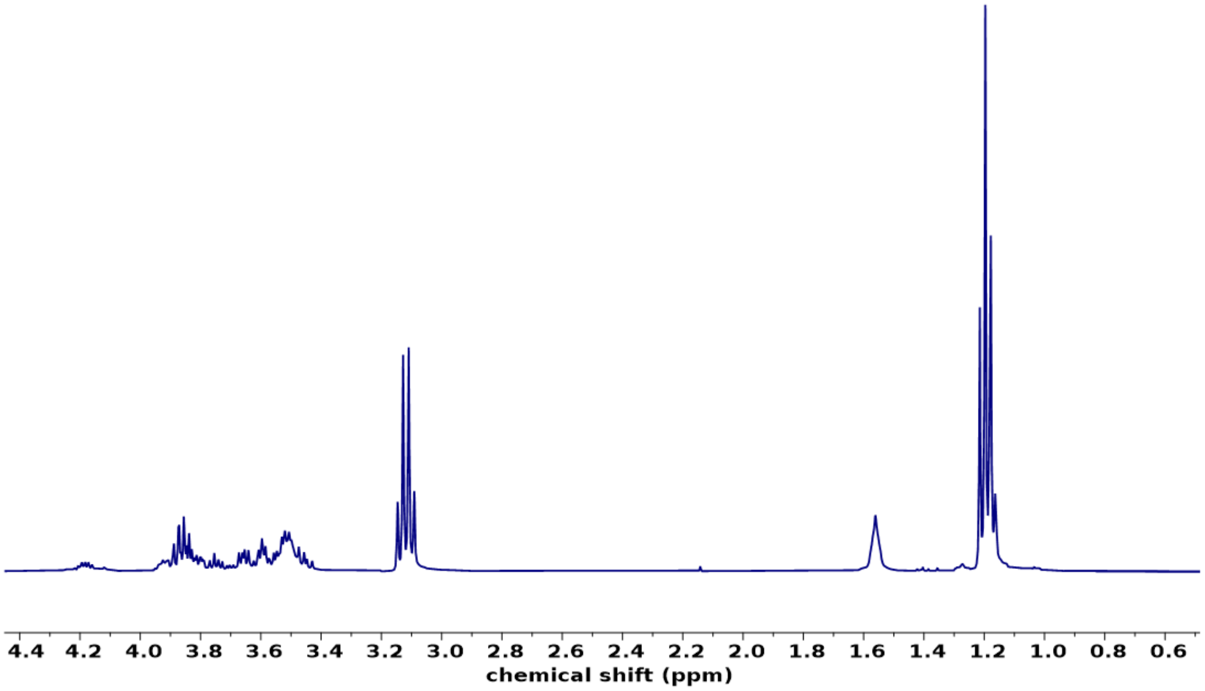


*Figure S8: $^{1}H$ NMR spectrum of **BDDE-dP-TEA** (400 MHz, $D_2O$) δ 4.23 – 4.14 (m, 1H), 4.01 – 3.37 (m, 17H), 3.12 (q, J = 7.3 Hz, 12H), 1.56 (dt, J = 3.6, 3.3 Hz, 4H), 1.16 (dt, 24H). Due to the presence of multiple isomers, peaks could not be unambiguously assigned.*

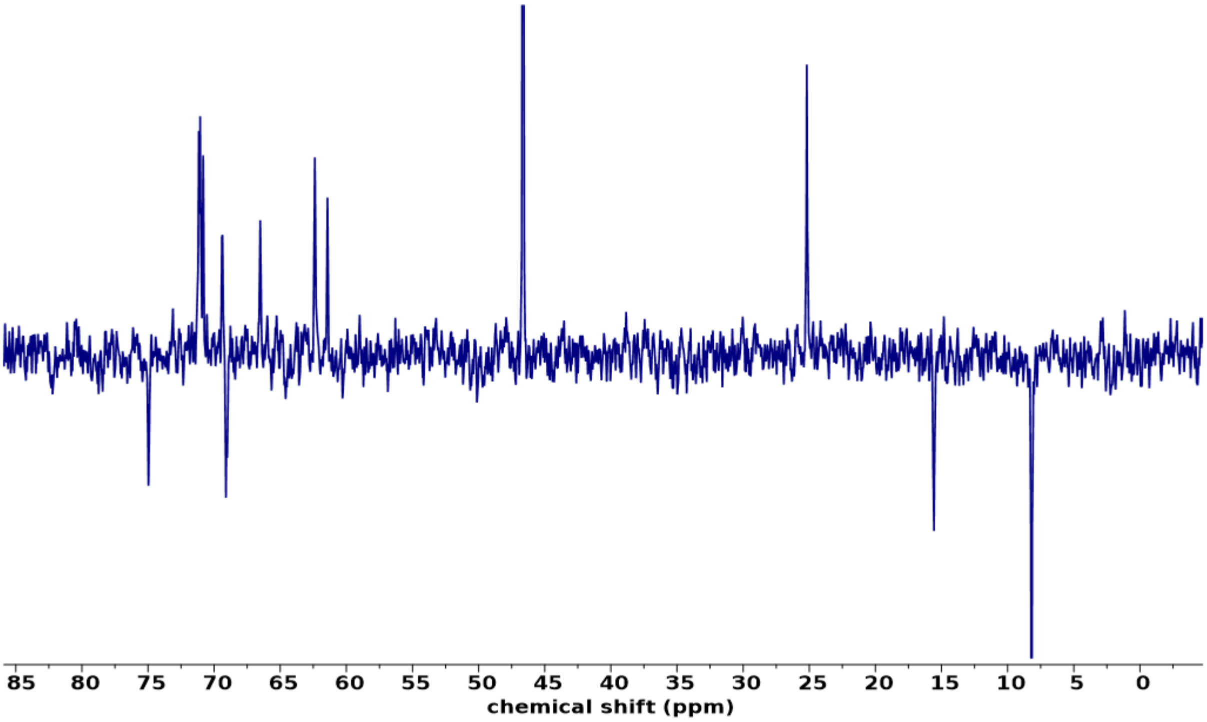


*Figure S9: $^{13}C$ NMR (attached proton test; C/$CH_2$ up, CH/$CH_3$ down) spectrum of **BDDE-dP-TEA** (101 MHz, $D_2O$) δ 74.97, 71.16, 71.06, 70.83, 69.37, 69.11, 69.03, 66.51, 62.38, 61.44, 46.65, 25.19, 15.57, 8.20. Due to the presence of multiple isomers, peaks could not be unambiguously assigned.*

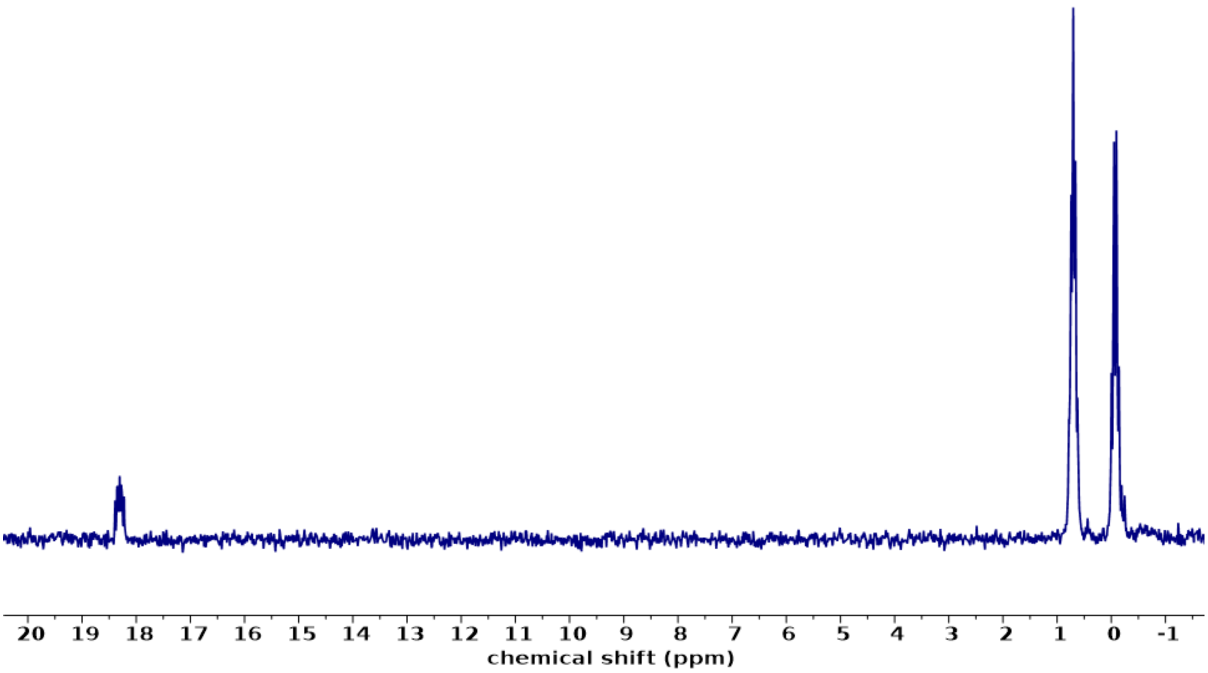


*Figure S10: $^{31}$P NMR spectrum of* ***BDDE-dP-TEA*** *(162 MHz, $D_2O$) δ 18.26 (m, J = 13.7 Hz), 0.70 (p, J = 6.3 Hz), -0.07 (q, J = 7.4 Hz).*

## Stress relaxation experiments

### *Normalized data and Arrhenius fit*

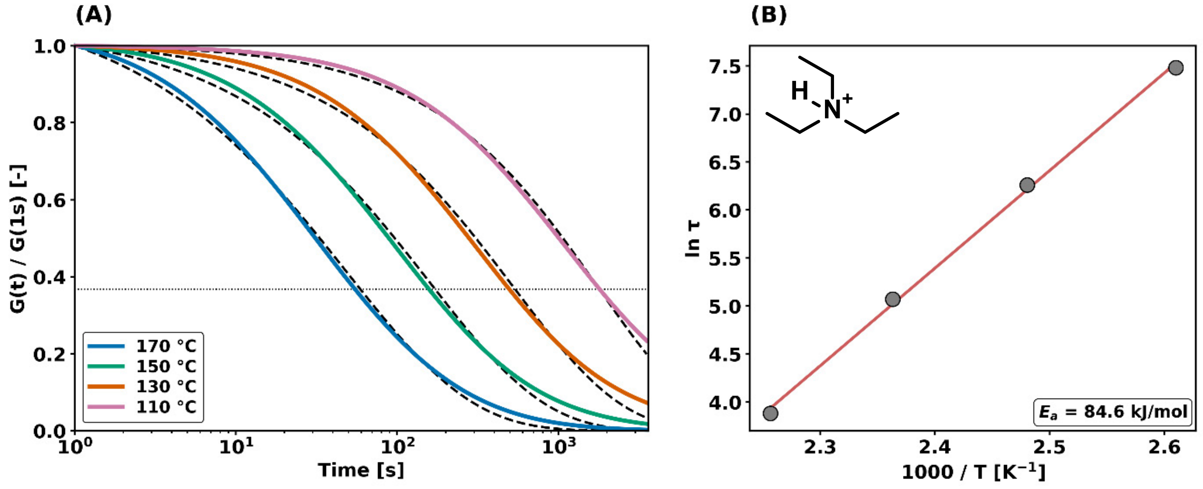


*Figure S11: Stress relaxation experiments on* ***PEG-dP-TEA****. (a) Normalized stress relaxation data on different temperatures. (b) Arrhenius plot using characteristic relaxation times derived from (a).*

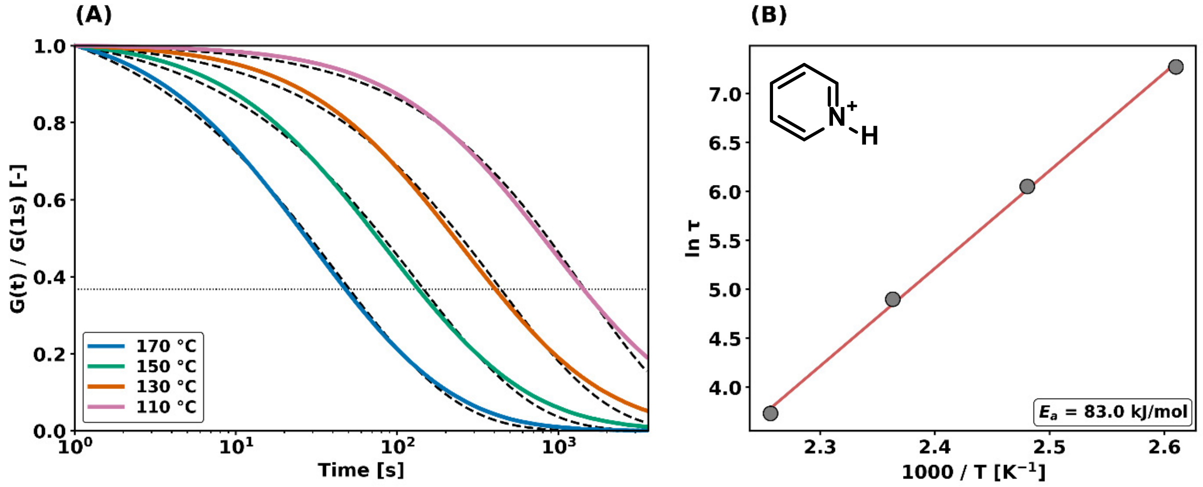


*Figure S12: Stress relaxation experiments on* ***PEG-dP-Pyr****. (a) Normalized stress relaxation data on different temperatures. (b) Arrhenius plot using characteristic relaxation times derived from (a).*

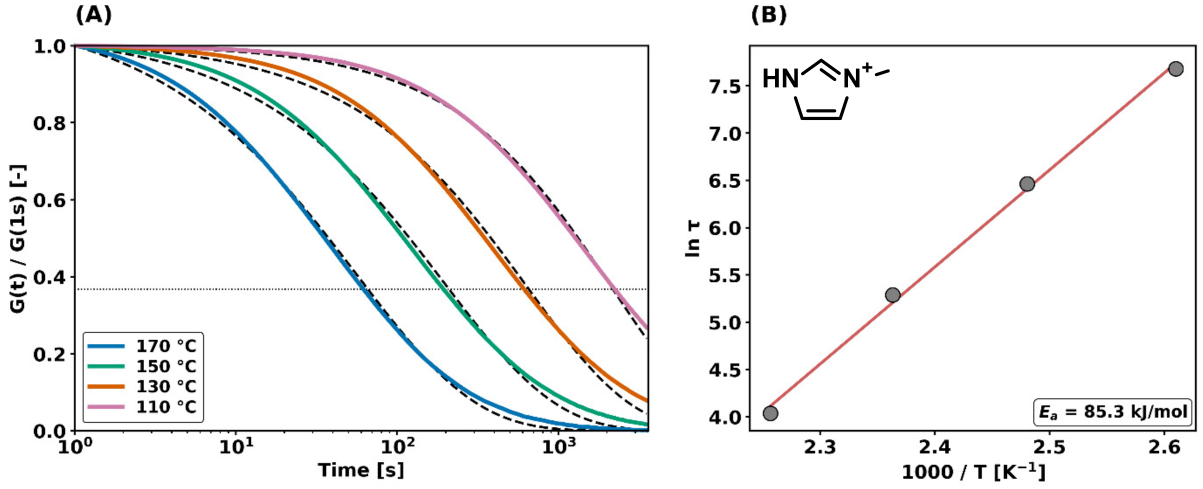


*Figure S13: Stress relaxation experiments on **PEG-dP-MIM**. (a) Normalized stress relaxation data on different temperatures. (b) Arrhenius plot using characteristic relaxation times derived from (a).*

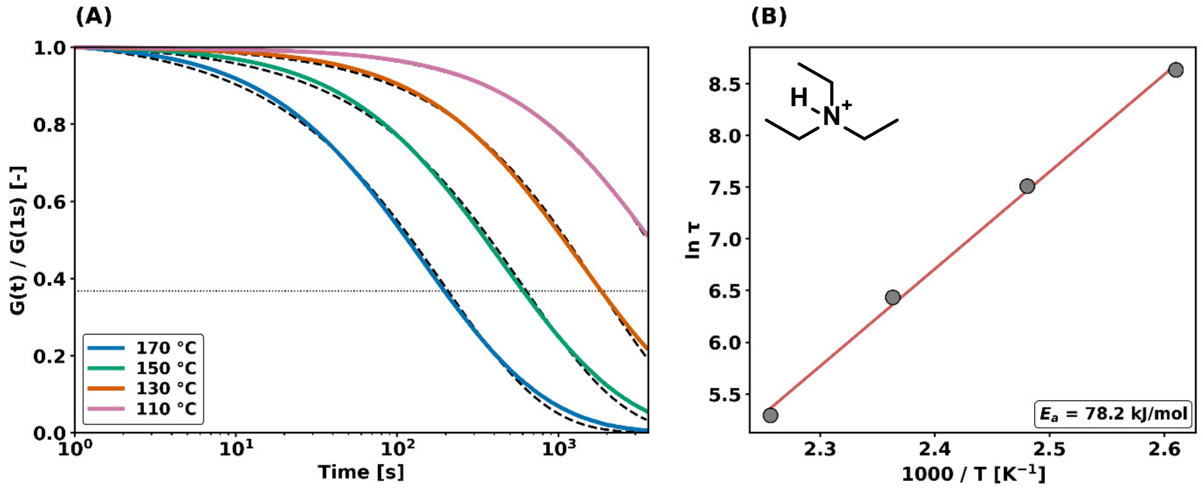


*Figure S14: Stress relaxation experiments on **PCL-dP-TEA**. (a) Normalized stress relaxation data on different temperatures. (b) Arrhenius plot using characteristic relaxation times derived from (a).*

*Table S1: Characteristic relaxation times (τ) and stretch parameters (β) as derived from plotting the normalized stress relaxation data above with the stretched exponential function.*

| | **PEG-dP-TEA** | | **PEG-dP-Pyr** | | **PEG-dP-MIM** | | **PCL-dP-TEA** | |
|---|---|---|---|---|---|---|---|---|
| | **τ (s)** | **β (-)** | **τ (s)** | **β (-)** | **τ (s)** | **β (-)** | **τ (s)** | **β (-)** |
| 170 °C | 49 | 0.56 | 42 | 0.57 | 57 | 0.59 | 192 | 0.67 |
| 150 °C | 159 | 0.60 | 134 | 0.61 | 198 | 0.63 | 615 | 0.70 |
| 130 °C | 523 | 0.64 | 426 | 0.65 | 642 | 0.67 | 1817 | 0.74 |
| 110 °C | 1779 | 0.70 | 1441 | 0.72 | 2165 | 0.74 | 5697 | 0.79 |
| $E_a$ | 84.6 kJ/mol | | 83.0 kJ/mol | | 85.3 kJ/mol | | 78.2 kJ/mol | |

*Relaxation modes via Tikhonov regularization*

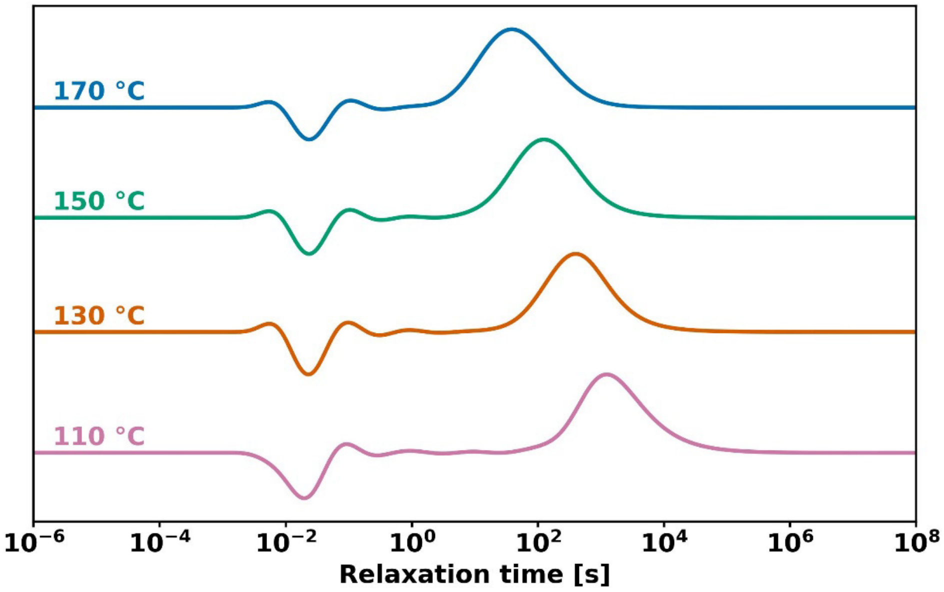


*Figure S15: Relaxation spectrum of stress relaxation on **PEG-dP-TEA** at different temperatures.*

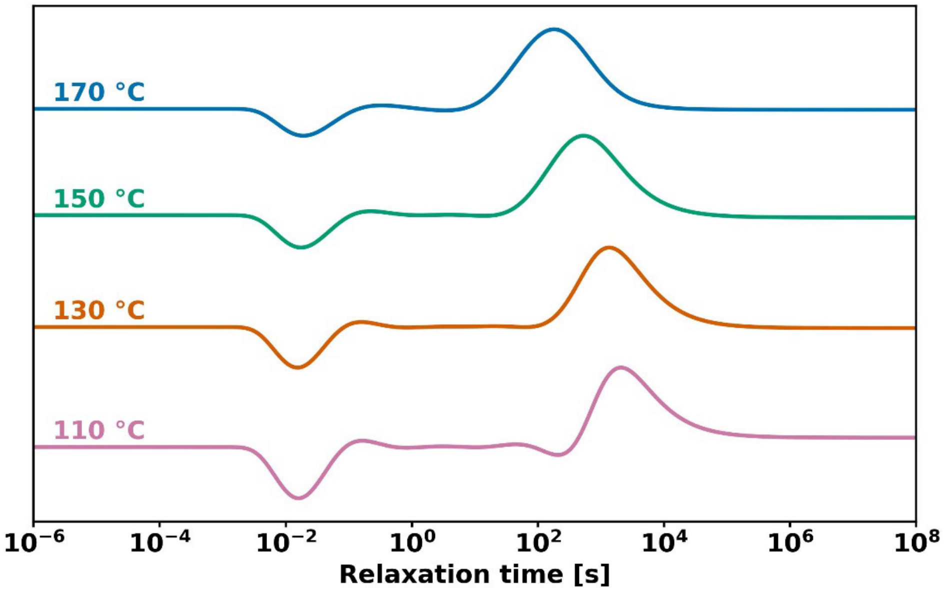


*Figure S16: Relaxation spectrum of stress relaxation on **PCL-dP-TEA** at different temperatures.*

*Repeated stress relaxation experiments*

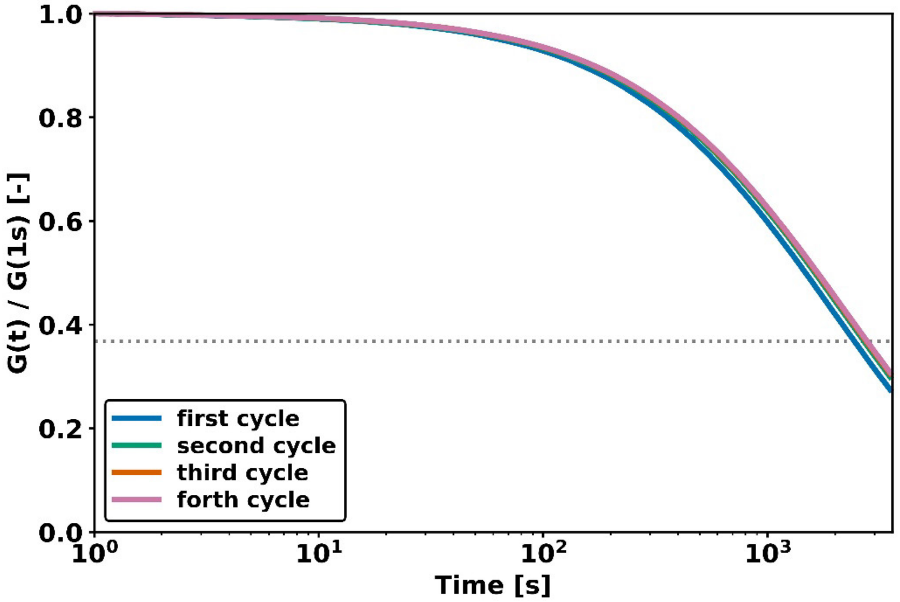


*Figure S17: Four cycles of repeated stress relaxation experiments on **PCL-dP-TEA** at 120 °C.*

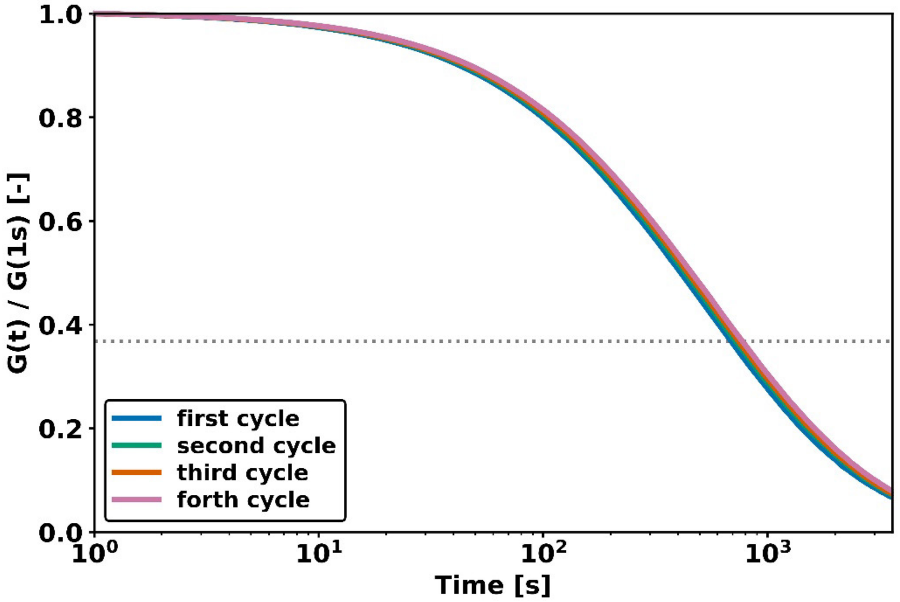


*Figure S18: Four cycles of repeated stress relaxation experiments on **PCL-dP-TEA** at 140 °C.*

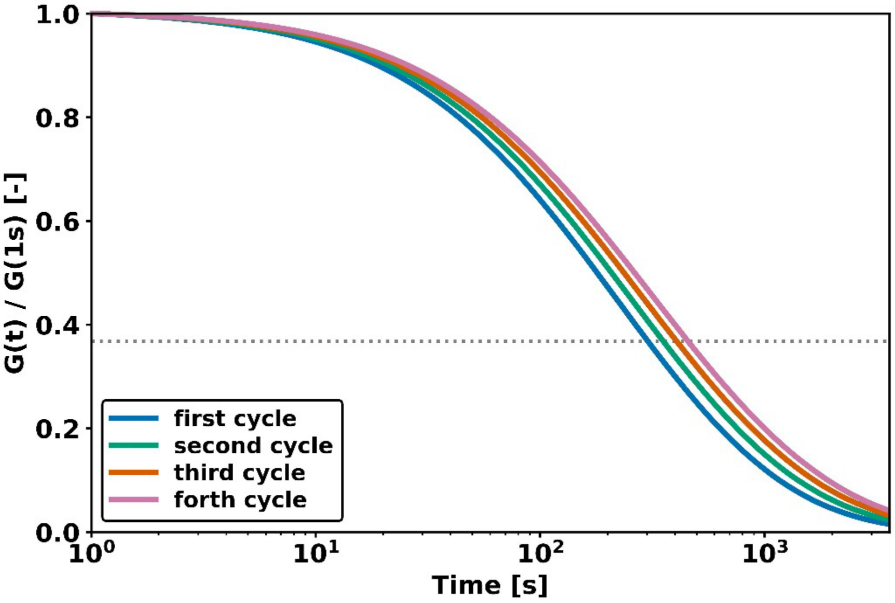


*Figure S19: Four cycles of repeated stress relaxation experiments on* ***PCL-dP-TEA*** *at 160 °C.*

Dynamic mechanical thermal analysis

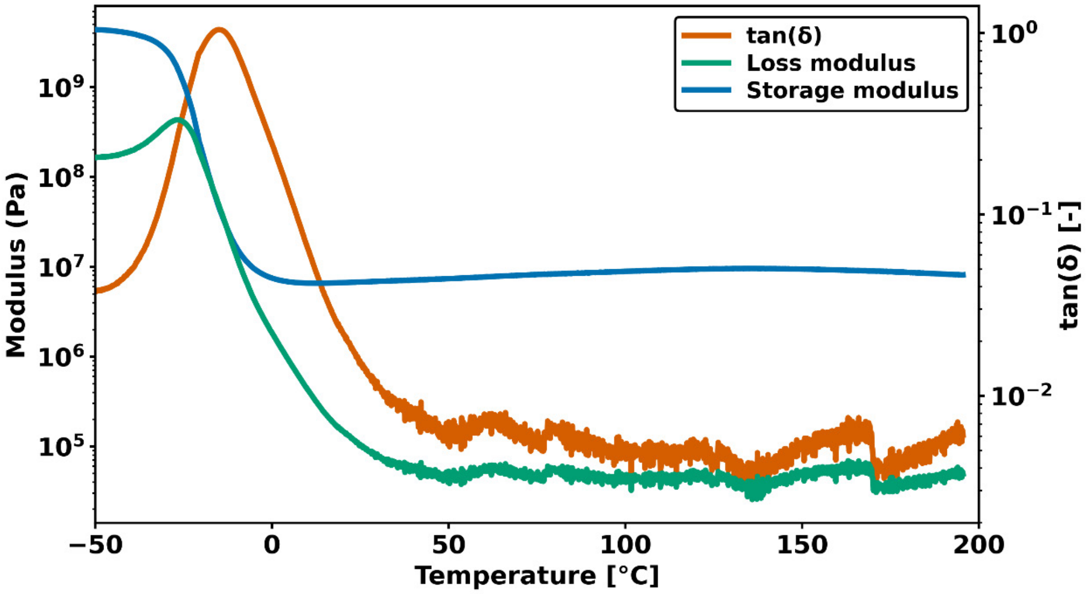


*Figure S20: DMTA profile for* ***PEG-dP-TEA****.*

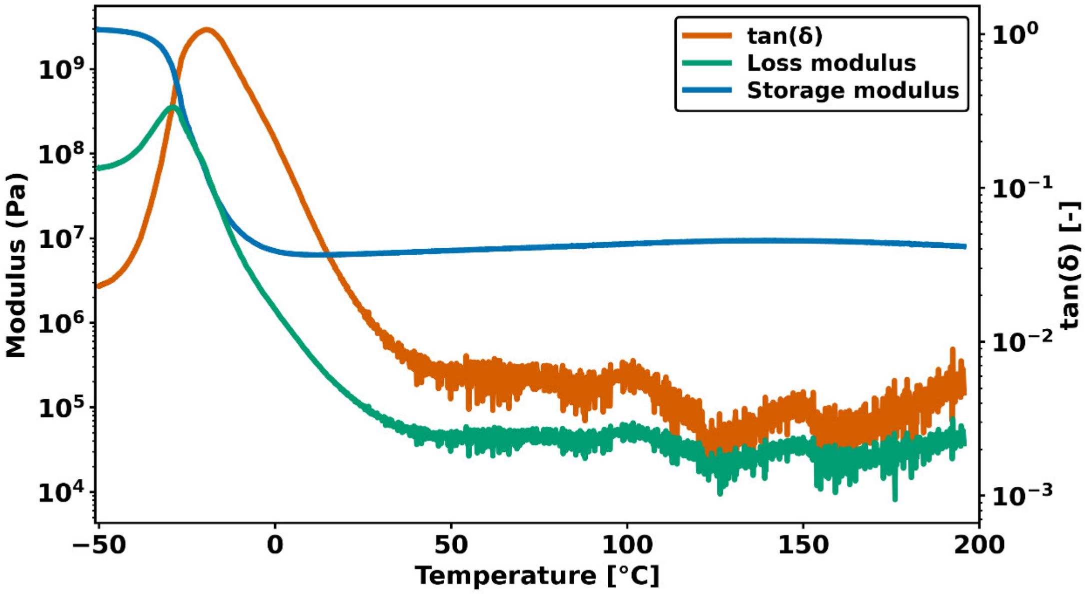


*Figure S21: DMTA profile for* ***PEG-dP-Pyr****.*

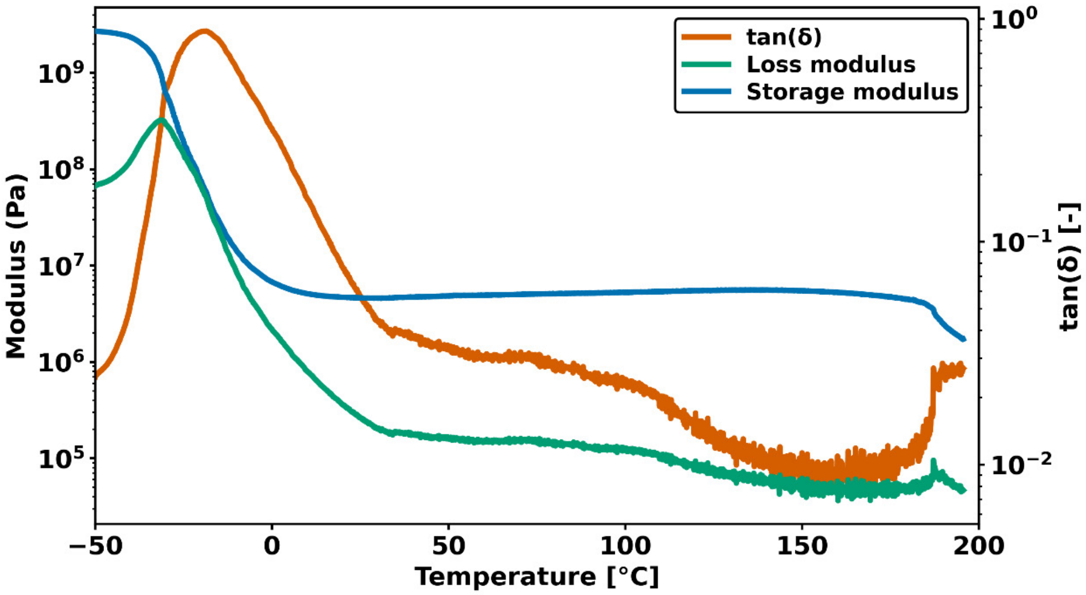


*Figure S22: DMTA profile for* ***PEG-dP-MIM****.*

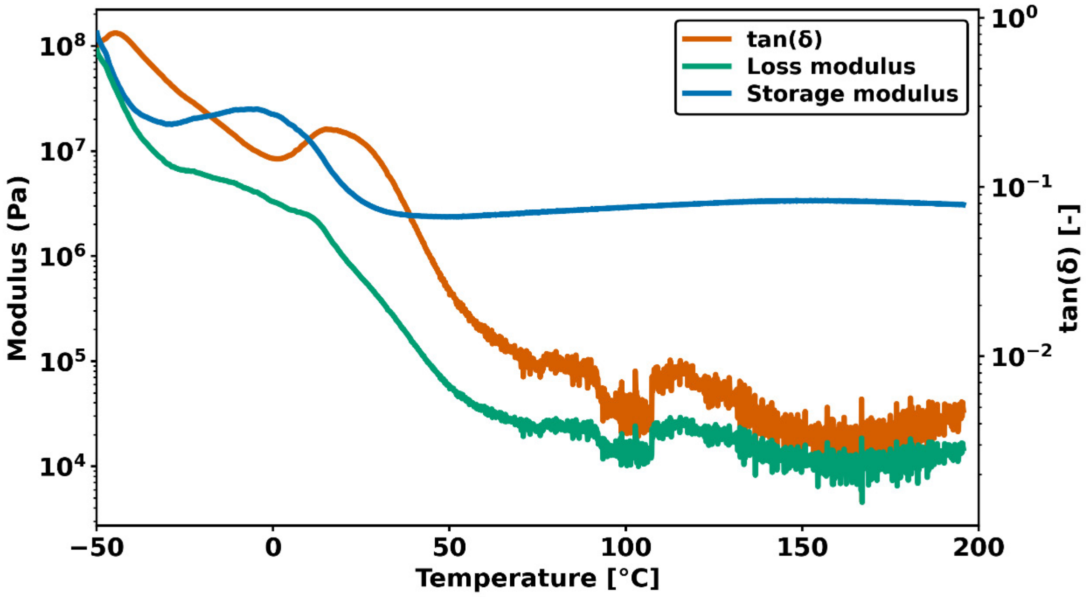


*Figure S23: DMTA profile for* ***PCL-dP-TEA****.*

Differential scanning calorimetry

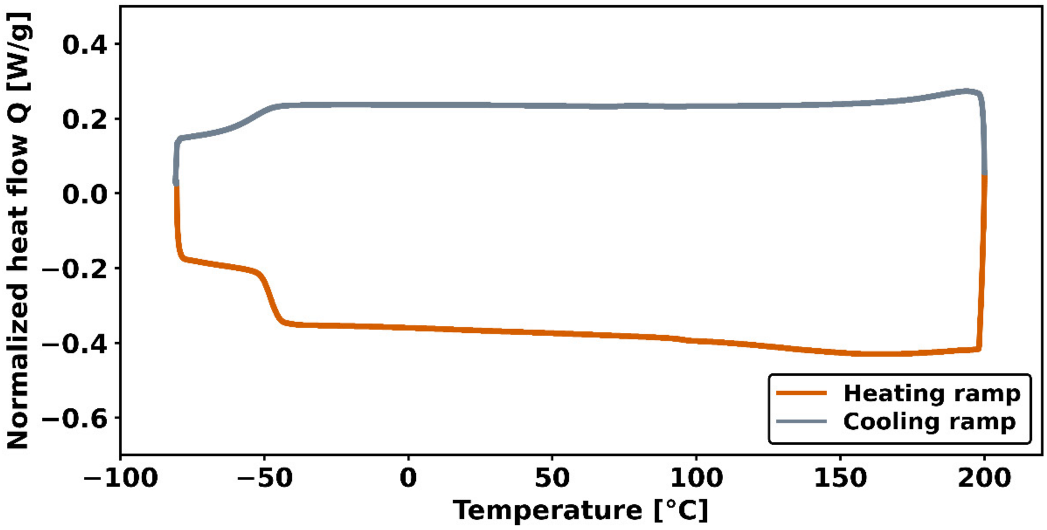


*Figure S24: DSC thermogram of the second heating and cooling run for* ***PEG-dP-TEA*** *(exo up).*

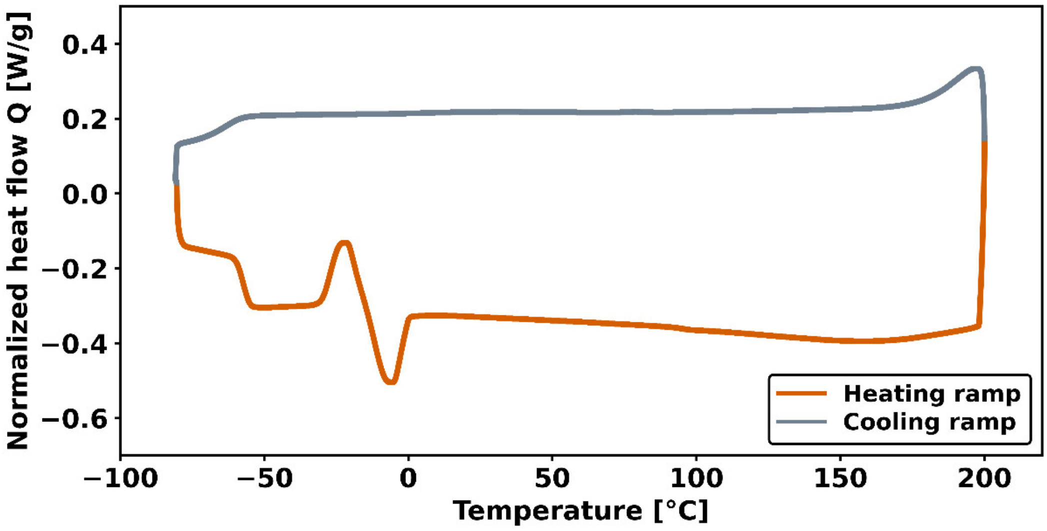


*Figure S25: DSC thermogram of the second heating and cooling run for* ***PEG-dP-Pyr*** *(exo up).*

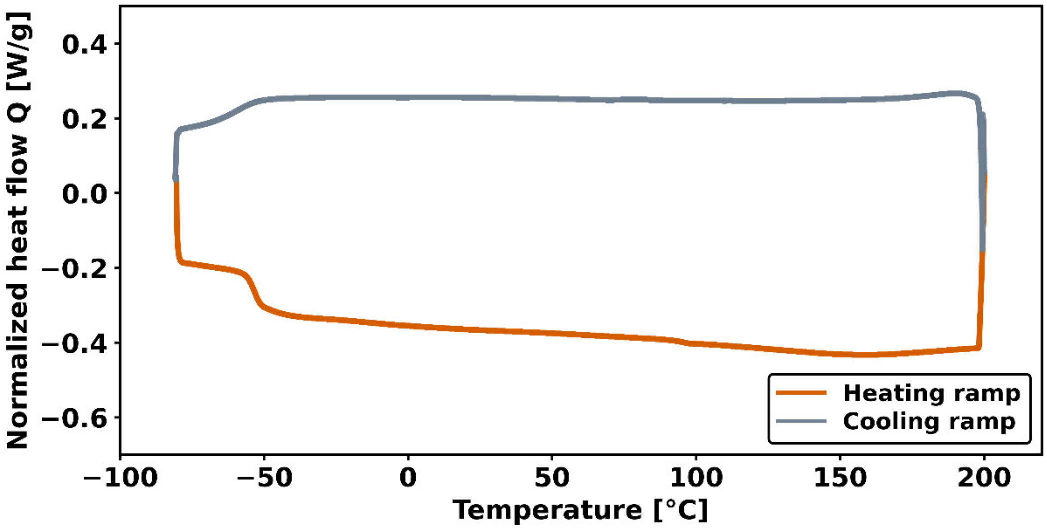


*Figure S26: DSC thermogram of the second heating and cooling run for* ***PEG-dP-MIM*** *(exo up).*

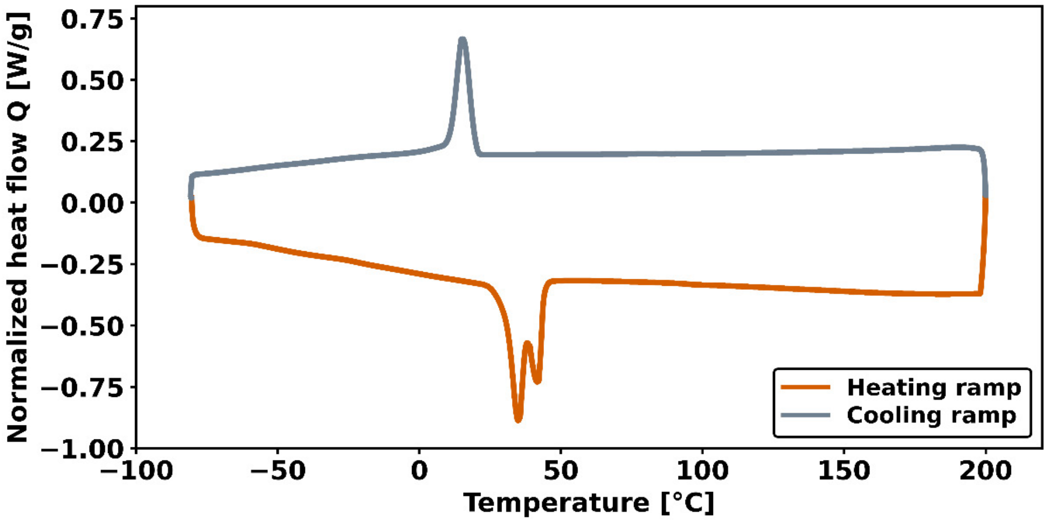


*Figure S27: DSC thermogram of the second heating and cooling run for* ***PCL-dP-TEA*** *(exo up).*

VT $^{31}P$ SSNMR

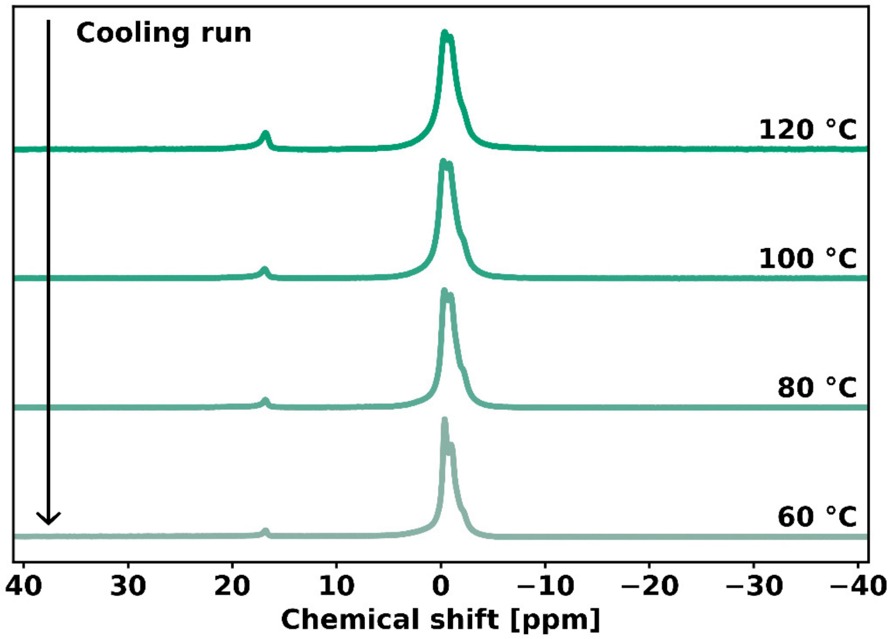


*Figure S28: cooling run of VT $^{31}P$ SSNMR measurements on* ***PEG-dP-TEA****.*

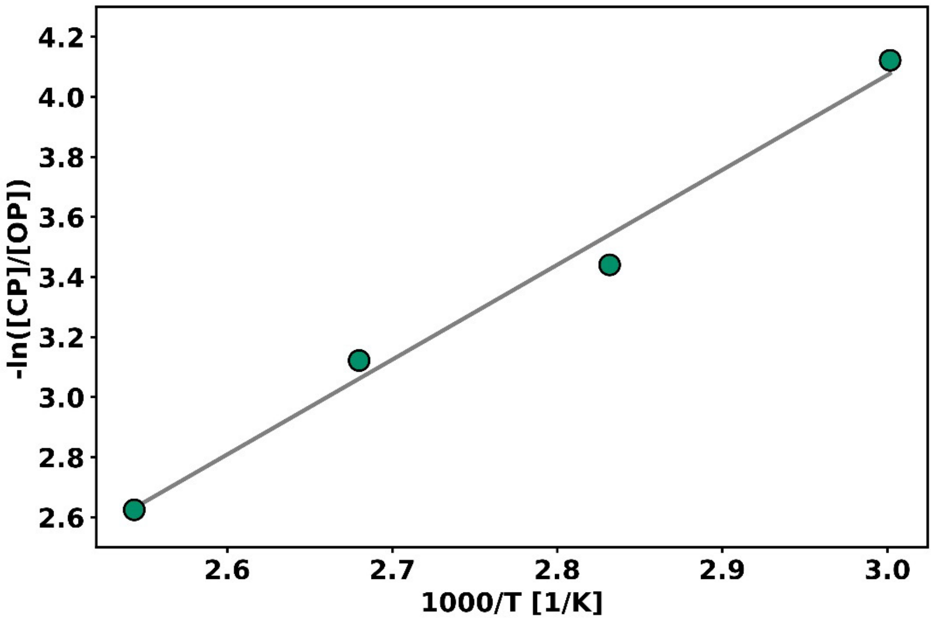


*Figure S29: Van 't Hoff plot as derived from data of the heating run of VT $^{31}P$ SSNMR measurements on* ***PEG-dP-TEA****.*

*Table S2: Ratios between [OP] and [CP] during heating and cooling cycles of VT $^{31}P$ SSNMR experiments on **PEG-dP-TEA**. All normalized to [CP]*

| | **Heating cycle** | | **Cooling cycle** | |
|---|---|---|---|---|
| | **[CP]** | **[OP]** | **[CP]** | **[OP]** |
| **60 °C** | 1 | 61.7 | 1 | 26.1 |
| **80 °C** | 1 | 31.1 | 1 | 27.3 |
| **100 °C** | 1 | 22.7 | 1 | 22.1 |
| **120 °C** | 1 | 13.8 | 1 | 13.8 |

# Supporting references


1 S. Majumdar, B. Mezari, H. Zhang, J. van Aart, R. A. T. M. van Benthem, J. P. A. Heuts and R. P. Sijbesma, *Macromolecules*, 2021, **54**, 7955-7962

2 J. Fukal, O. Páv, M. Buděšínský, J. Šebera and V. Sychrovský, *Phys. Chem. Chem. Phys.*, 2017, **19**, 31830–31841.

3 R. Bodalski and L. D. Quin, *J. Org. Chem.*, 1991, **56**, 2666–2671.

4 V. Kontogiorgos, in *Biopolymers*, IntechOpen, 2010.

5 P. C. Hansen, *Numer. Algorithms*, 1994, **6**, 1–35.
